# Harpagoside attenuates local bone Erosion and systemic osteoporosis in collagen-induced arthritis in mice

**DOI:** 10.1186/s12906-022-03694-y

**Published:** 2022-08-10

**Authors:** Ju-Young Kim, Yoon-Hee Cheon, Sung-Jun Ahn, Sung Chul Kwak, Chong Hyuk Chung, Chang Hoon Lee, Myeung Su Lee

**Affiliations:** 1 Musculoskeletal and Immune Disease Research Institute, School of Medicine, Wonkwang University, 460 Iksandae-ro, Iksan, Jeonbuk 54538 Republic of Korea; 2 Division of Rheumatology, Department of Internal Medicine, Wonkwang University Hospital, 460 Iksandae-ro, Iksan, Jeonbuk 54538 Republic of Korea

**Keywords:** Collagen-induced arthritis model, Harpagoside, Inflammation, Osteoclasts, Rheumatoid arthritis

## Abstract

**Background:**

Rheumatoid arthritis (RA) is a chronic inflammatory autoimmune disease that causes local bone erosion and systemic osteoporosis. Harpagoside (HAR), an iridoid glycoside, has various pharmacological effects on pain, arthritis, and inflammation. Our previous study suggests that HAR is more deeply involved in the mechanism of bone loss caused by inflammatory stimuli than hormonal changes. Here, we identified the local and systemic bone loss inhibitory effects of HAR on RA and its intracellular mechanisms using a type 2 collagen-induced arthritis (CIA) mouse model.

**Methods:**

The anti-osteoporosis and anti-arthritic effects of HAR were evaluated on bone marrow macrophage in vitro and CIA in mice in vivo by obtaining clinical scores, measuring hind paw thickness and inflammatory cytokine levels, micro-CT and histopathological assessments, and cell-based assay.

**Results:**

HAR markedly reduced the clinical score and incidence rate of CIA in both the prevention and therapy groups. Histological analysis demonstrated that HAR locally ameliorated the destruction of bone and cartilage and the formation of pannus. In this process, HAR decreased the expression of inflammatory cytokines, such as tumor necrosis factor-α, interleukin (IL)-6, and IL-1β in the serum of CIA mice. Additionally, HAR downregulated the expression of receptor activator of nuclear factor-κB ligand and upregulated that of osteoprotegerin. HAR suppressed systemic bone loss by inhibiting osteoclast differentiation and osteoclast marker gene expression in a CIA mouse model.

**Conclusions:**

Taken together, these findings show the beneficial effect of HAR on local symptoms and systemic bone erosion triggered by inflammatory arthritis.

**Supplementary Information:**

The online version contains supplementary material available at 10.1186/s12906-022-03694-y.

## Background

Rheumatoid arthritis (RA) is an autoimmune disease that is accompanied by chronic synovitis, progressive cartilage, and bone destruction and is affected by inflammatory-associated factors, including multiple cytokines and inflammatory factors in the erosion region [1, 2]. The pathophysiological mechanisms and causes of RA remain unclear, it is known that various immune cells including T- and B-lymphocytes, osteoclasts, fibroblast-like synoviocytes, and chondrocyte are involved in auto-immunity and chronic inflammation during RA pathogenesis. In particular, osteoclasts play a crucial role in cartilage and bone destruction in RA. Since osteoclasts act as unique effector cells in RA, the regulation of osteoclast differentiation and function could be an important target for RA treatment. Therefore, many studies have reported the crosstalk between osteoclast differentiation and the immune response in RA [3–5].

One of the notable features of RA is bone erosion in the joint region that is related to disease severity and poor functional outcomes. Multiple pro-inflammatory cytokines, such as receptor activator of NF-κB ligand (RANKL), tumor necrosis factor (TNF)-α, interleukin (IL)-1, and IL-6, are involved in cartilage and bone destruction. The main causes of joint bone erosion are synovitis, including the production of inflammatory cytokines and RANKL, and antibody response to citrullinated proteins [4, 5]. RANKL binds to RANK in osteoclast precursors and mature osteoclasts, consequently initiating downstream signaling and accelerating osteoclast differentiation and bone resorption. RANKL-deficient mice recovered from bone erosion and cartilage destruction in the serum transfer model of arthritis [6]. As an inhibitor of RANKL, denosumab improves bone loss in osteoporosis with RA [7, 8]. However, denosumab is not widely used for the treatment of RA or concomitant osteoporosis. Therefore, it is necessary to establish the optimal treatment through additional studies, including clinical studies using denosumab [9]. Also, current treatment strategies for RA focus on ameliorating joint damage and inflammatory responses including swelling and fever, with glucocorticoids, specific inhibitors of inflammatory cytokines, and nonsteroidal anti-inflammatory drugs (NSAIDs) [10]. However, these treatment are reported to cause side effects on the cardiovascular system, kidneys, and liver, making it difficult to select a treatment method. The main side effects of glucocorticoids are gastrointestinal ulceration and bleeding, infection, immunosuppression, and bone damage [11]. It has been reported that side effects of specific inhibitors of inflammatory cytokines, such as anti-TNF biologics, increase infections such as tuberculosis and pneumonia [12], and increase the risk of malignancy [13] and nervous system [14]. Also, the side effects of NSAIDs inhibit platelet aggregation and cause serious gastrointestinal disorders such as bleeding, ulceration and perforation and renal toxicity [15], and also adversely affect the cardiovascular system, including congestive heart failure, acute myocardial fraction, and even sudden death [16]. Therefore, it has become essential to find natural products that are safe and effective in the treatment of RA.

Harpagoside (HAR) is a natural compound obtained from medicinal plants, such as *Harpagophytum procumbens (devil’s claw), Scrophularia ningpoensis, Scrophularia buergeriana, and Harpagophytum procumbens* [17–19]. It has traditionally been used as a treatment for arthritis, cancer, immune diseases, inflammatory diseases, and pain relief [20, 21]. In particular, bone loss can be reduced by controlling osteoclast differentiation and function [22]. We previously demonstrated that HAR attenuated inflammation-mediated bone loss but not ovariectomized bone loss in a mouse model [22]. Overall, HAR might be more deeply involved in the mechanism of bone loss caused by inflammatory stimuli rather than hormonal changes. Here, we identified the underlying mechanism by which HAR prevented and treated RA and arthritis-induced bone erosion by focusing on osteoclast differentiation using in vitro and in vivo experiments.

In this study, we demonstrated the inhibitory effect of HAR on synovial inflammation, joint destruction, and bone erosion in collagen-induced arthritis (CIA) mice models. In addition, the efficacy of HAR according to local and systemic applications in vivo was verified separately. In vitro, we confirmed the effect of HAR on osteoclast differentiation and function through an underlying mechanism based on the RANK-RANKL system.

## Materials and methods

### Reagents

HAR (purity >95%) was purchased form Sigma-Aldrich (St. Louis, MO, USA) and dissolved in dimethyl sulfoxide (DMSO, Sigma-Aldrich) to make 100 mM stock solution and stored at −20 °C.

### Animals

Twenty pathogen-free male DBA/1 mice at 8 weeks of age were purchased from Samtako (Osan, Korea). The mice were housed in a temperature (22–24 °C) and humidity (55–60%) controlled environment with a 12-h light/dark cycle and were maintained on standard laboratory chow ad libitum. All experiments were performed in accordance with the guidelines for animal experimentation of the Institute Committee of Wonkwang University (WKU15–78).

### Induction of CIA in DBA/1 mice

**Fig. 1:**
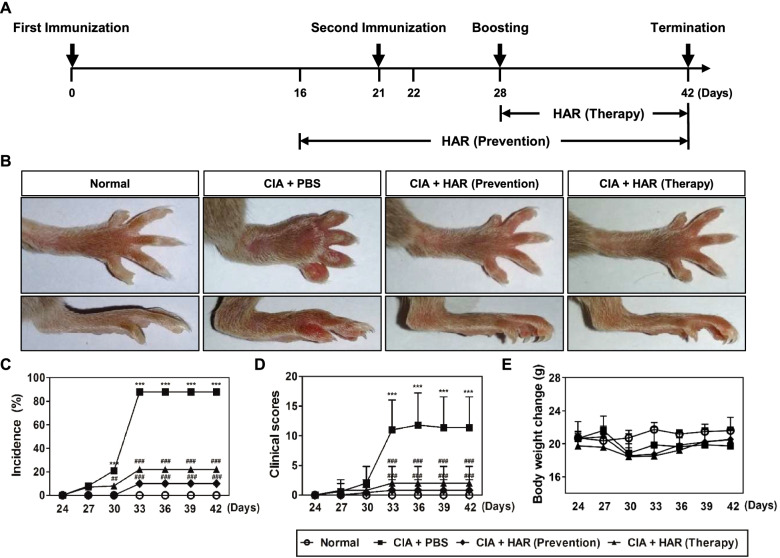
Effect of harpagoside (HAR) on the progression of collagen-induced arthritis (CIA). **A** Study design to induce CIA in mice. Eight-week-old male DBA/1 mice were first immunized with Col 2 and CFA on day 0 and then immunized intraperitoneally with Col 2 on day 21. Next, booster injection with LPS was given on day 28. CIA mice were orally administrated with HAR (10 mg/kg) every day from day 16 to 42 in the prevention group and from day 28 to 42 in the therapy group. **B** Representative photographs of normal, PBS-, and HAR-treated CIA mice. **C** The incidence of arthritis, **D** clinical scores, and **E** body weight change were determined on the indicated days. ^***^p < 0.001 versus the normal group; ^*##*^*p* < 0.01 and ^*###*^*p* < 0.001 versus the CIA + PBS group

DBA/1 mice were immunized with 150 μL of bovine type II collagen (CII) emulsified with an equal volume of complete Freund’s adjuvant (CFA; Chondrex, Redmond, WA, USA). The point of initial immunization was designated day 0. The mice were then boosted with an equal amount of bovine type II collagen emulsified in Freund’s incomplete adjuvant (IFA; Chondrex) on day 21 and by an intraperitoneal injection of lipopolysaccharide (LPS, 30 μg; Sigma-Aldrich, St. Louis, MO, USA) was on day 28 [23]. To evaluate the preventive or therapeutic effect of HAR on CIA progression, HAR (10 mg/kg) or PBS was orally administered every other day. The experiment consisted of four groups: group 1, control with no disease (*n* = 5); group 2, CIA with vehicle treatment (*n* = 5); group 3, CIA with HAR treatment at 5 days before second immunization (Prevention, *n* = 5); group 4, CIA with HAR treatment at 1 day after the second immunization (Therapy, *n* = 5) (Fig. 1A). The mice were monitored daily in a blinded manner for signs of arthritis. The symptoms were graded and scored as previously described [23]. Briefly, all four limbs of the mice were evaluated and scored from 0 to 4 according to the following scale: 0 = no swelling; 1 = slight swelling and erythema confined to either the ankle or midfoot; 2 = slight swelling extending from the ankle to midfoot; 3 = moderate swelling from the ankle to metatarsal joints; and 4 = severe swelling in the ankle, foot, and digits.

### Micro-computed tomography (micro-CT) analyses

Micro-CT images of the hind paw and femur of the mice in all four groups were acquired on day 42, using a high-resolution micro-CT (NFR-Polaris-S160; Nanofocus Ray, Iksan, Korea) with a source voltage of 45 kVp, current of 90 μA, and an isotropic resolution of 133 mA. To verify bone destruction, 3-dimensional models of the hind paw and calcaneus and 2-dimensional models of the femur were reconstructed using INFINITT-Xelis software (INFINITT Healthcare, Seoul, Korea). The structural parameters included total porosity, trabecular pattern factor (Tb·Pf), trabecular bone volume/total volume (BV/TV, %), trabecular thickness (Tb·Th, mm), trabecular separation (Tb·Sp, mm), and trabecular number (Tb·N, 1/mm).

### Histopathological assessment

Joint tissues and femurs were fixed with 10% formalin for 24 h, decalcified for 3 weeks in 12% EDTA, and then embedded in paraffin. Section slides (5 μm thick) of ankle joints prepared using a Leica microtome RM2145 (Leica Microsystems, Bannockburn, IL, USA) were stained with hematoxylin and eosin (H&E), safranin O, or toluidine blue. The histological arthritis score was determined in a blinded fashion for changes in synovial proliferation, inflammation, cartilage damage, and bone erosion and scored on a scale of 0–4 [15]. To investigate the effect of osteoclasts on bone erosion in CIA mice, tissue sections were stained with tartrate-resistant acid phosphatase (TRAP). The number of TRAP-positive cells per field of tissue were quantified using Image Pro-Plus software version 4.0 (Media Cybernetics, Silver Spring, MD, USA).

### Human synovial cell culture and treatment

Human synovial cells SW982 were purchased from American Type Culture collection and maintained in DMEM with 10% fetal bovine serum (FBS) and 1% penicillin-streptomycin (P/S) in a humidified atmosphere with 5% CO_2_ at 37 °C. For the experiments, cells were pretreated various doses of HAR (0, 25, 50 and 100 μM) for 1 h and then incubated with TNFα (50 ng/mL) for 24 h.

### Preparation of mouse bone marrow macrophages (BMMs) and osteoclast differentiation

Bone marrow cells (BMCs) from 5-week-old ICR mice were isolated by flushing the femurs and tibiae with α-minimum essential medium (α-MEM) and then suspended in α-MEM supplemented with 10% FBS and 1% P/S as described previously [16]. The suspended BMCs were culted in the presence of macrophage colony-stimulating factor (M-CSF, 10 ng/mL) for 1 day in 10 cm culture dishes. Non-adherent cells were transferred to 10 cm non-tissue culture-treated petri dishes and then cultured for 3 days in the presence of M-CSF (30 ng/mL). Floating cells were discarded, and cells adhering to the bottom of the culture dish were classified as BMMs. BMMs were seeded at 3.5 × 10^4^ cells/well and cultured in the presence of M-CSF (30 ng/mL) and RANKL (50 ng/mL) for 4 days in the presence of various concentrations of HAR. The cells were fixed in 3.7% formalin for 10 min, permeabilized with 0.1% Triton X-100, and stained with TRAP solution. TRAP-positive multinucleated cells with more than five nuclei were counted as osteoclasts.

### Cell viability assay

SW982 were seeded in 96-well plates and incubated for 24 h. The cells were pretreated various doses of HAR (0, 25, 50 and 100 μM) for 1 h and then incubated with TNFα (50 ng/mL) for 24 h. After incubation for 3 days, the XTT solution (50 μL) was added to each well and incubated for 2 h. The plate was read at 450 nm using an ELISA reader (Molecular Devices, CA, USA). Four replicates were assessed for each sample.

### Pit formation assay

BMCs (1 × 10^7^ cells) and primary osteoblasts (1 × 10^6^ cells) were seeded on collagen gel-coated culture dishes and cultured for 7 days in the presence of 10^−8^ M 1,25-dihydroxyvitamin D3 (Sigma-Aldrich) and 10^−6^ M prostaglandin E2 (Sigma-Aldrich) as described previously [22]. The co-cultured cells were detached by 0.1% collagenase treatment at 37 °C for 10 min and then re-plated on hydroxyapatite-coated plates (Corning, Corning, NY, USA). The cells were incubated in the presence of various concentrations of HAR. After 12 h, the cells were removed, and the total resorption pits were photographed and analyzed using Image-Pro Plus version 4.0 (Media Cybernetics, Silver Spring, MD, USA).

### Quantitative real-time reverse transcription polymerase chain reaction (real-time RT-PCR) analyses

Total RNA was isolated using QIAzol reagent (QIAGEN, Valencia, CA, USA) following the manufacturer’s instructions. To obtain cDNA, equal amounts of total RNA were reverse-transcribed into cDNA using the Reverse Transcription System cDNA synthesis kit (Promega, Madison, WI, USA). Real-time RT-PCR was performed in a 20 μL reaction mixture containing 10 μL of SYBR Green Premix (Bioneer Co., Daejeon, Korea), 10 pmol of forward primer, 10 pmol of reverse primer, and 1 μg of cDNA using an Exicycler™ 96 Real-Time Quantitative Thermal Block (Bioneer Co.) as described previously [24]. The follwing mouse-specific primers used: *mouse TRAP*, forward 5′-TCATGGGTGGTGCTGCT-3′ and reverse 5′-GCCCACAGCCACAAATCT-3′; *mouse osteoclast-associated receptor* (*OSCAR*), forward 5′-GGAATGGTCCTCATCTGCTT-3′ and reverse 5′-GGAATGGTCCTCATCTGCTT-3′; *mouse calcitonin receptor* (*CTR*), forward 5′-TCCAACAAGGTGCTTGGGAA-3′ and reverse 5′-CTTGAACTGCGTCCACTGGC-3′; *mouse Cathepsin K*, forward 5′-CACTGCTCTCTTCAGGGCTT-3′ and reverse 5′-ACGGAGGCATTGACTCTGAA-3′; *mouse glyceraldehyde-3-phosphate dehydrogenase* (*GAPDH*), forward 5′-TCAAGAAGGTGGTGAAGCAG-3′ and reverse 5′-AGTGGGAGTTGCTGTTGAAGT-3′; human *RANKL*, forward 5′-GTGCAAAAGGAATTACAACATATCGT-3′ and reverse 5′-AACCATGAGCCATCCACCAT-3′; human *TNF-α*, forward 5′-CCCCAGGGACCTCTCTCTAATC-3′ and reverse 5′-GGTTTGCTACAACATGGGCTACA-3′; human *IL-6*, forward 5′- AACCTGAACCTTCCAAAGATGG-3′ and reverse 5′-TCTGGCTTGTTCCTCACTACT-3′; human *IL-1β*, forward 5′-ATGATGGCTTATTACAGTGGCAA-3′ and reverse 5′-ATGATGGCTTATTACAGTGGCAA-3′; and human *GAPDH*, forward 5′-AGAAGGCTGGGGCTCATTTG-3′ and reverse 5′-AGGGGCCATCCACAGTCTTC-3′. The mouse *GAPDH* gene was used as the internal control. The amplification parameters consisted of an initial denaturation step at 95 °C for 5 min, followed by 40 cycles of denaturation at 95 °C for 1 min, annealing at 60 °C for 30 s, and extension at 72 °C for 1 min. Expression data were analyzed using the 2^-ΔΔCT^ method.

### Western blot analyses

Whole-cell lysates were prepared using lysis buffer containing 50 mM Tris-HCl, 150 mM NaCl, 5 mM EDTA, 1% Triton X-100, 1 mM sodium fluoride, 1 mM sodium vanadate, 1% deoxycholate, and protease inhibitors as described previously [24]. Equal amounts of protein (20 μg) were run on 8–10% SDS-PAGE gels and were transferred by electroblotting onto polyvinylidene difluoride membranes (Millipore, Bedford, MA, USA). Non-specific interactions were blocked with 5% skim milk for 1 h, and the membranes were incubated for 2 h with the following primary antibodies: anti-p38 (Lot No. 23), anti-phospho-p38 (Lot No. 24), anti-JNK (Lot No. 17), anti-phospho-JNK (Lot No. 27), anti-Akt (Lot No. 28), anti-phospho-Akt (Lot No. 14), anti-ERK (Lot No. 26), anti-phospho-ERK (Lot No. 29), anti-IκB (Lot No. 11), anti-phospho-IκB (Lot No. 18), anti-phospho-p65-NF-κB (Lot No. 10) (Cell Signaling Technology, Beverly, MA, USA); anti-p65-NF-κB (Lot No. D2315), anti-c-Fos (Lot No. G0111), anti-NFATc1 (Lot No. E1011) (Santa Cruz Biotechnology, Santa Cruz, CA, USA); and β-actin (Lot No. SF253548) (Sigma-Aldrich). The membranes were washed in tris-buffered saline contacting 0.1% Tween-20 and incubated for 1 h with horseradish peroxidase-conjugated sheep anti-mouse (Lot No. 04132108) or donkey anti-rabbit (Lot No. 03092126) immunoglobulin antibodies (Enzo Life Sciences, New York, USA). Specific signals were detected using the Western Chemiluminescent HRP substrate kit (Millipore).

### Cytokine analyses

To determine cytokine levels in CIA mice, serum was obtained on day 42. Serum RANKL (Lot No. 321174), osteoprotegerin (OPG) (Lot No. 312709), TNF-α (Lot No. 893961), IL-6 (Lot No. 892369), and IL-1β (Lot No. 893829) were measured using an enzyme-linked immunosorbent assay (ELISA) kit (R&D systems, Minneapolis, MN, USA) according to the manufacturer’s guidelines.

### Statistical analysis

Each experiment was performed at least three times, and the data are expressed as the mean ± standard deviation (SD) or the mean ± standard error (SE). All data were analyzed by one-way ANOVA, followed by the multiple comparisons Tukey’s post-hoc test, using the Statistical Package for the Social Sciences software (SPSS; Korean version 14.0). A *p* value less than 0.05 was considered statistivally significant.

## Results

### HAR preventively and therapeutically reduces synovial inflammation, joint destruction, and local bone erosion in CIA mice

**Fig. 2:**
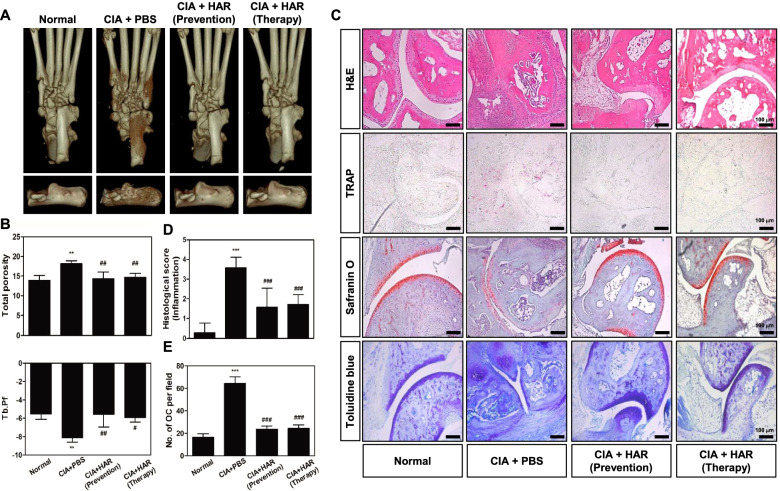
Effect of harpagoside (HAR) on the severity of arthritis and histopathological changes in collagen-induced arthritis (CIA) mice. On day 42 after the initial immunization with Col 2, systemic bone loss was analyzed by micro-CT, and bone destruction and cartilage damage of CIA mice were analyzed by histological staining of the ankle joint. **A** Representative 3D images of the hind paw (up) and calcaneus (down). **B** The total porosity, trabecular thickness (Tb.Th), and trabecular pattern factor (Tb.Pf) of the calcaneus. **C** The ankle joint was sectioned and stained with H&E, TRAP, Safranin O, and toluidine blue. Scale bar, 100 μm. **D** The pathological severity scores of inflammation, pannus formation, cartilage damage, and bone damage in the ankle joint of CIA mice. **E** The number of osteoclasts per field was counted using the histomorphometric methods. ^**^p < 0.01 and ^***^p < 0.001 versus the normal group; ^*#*^ *p* < 0.05, ^*##*^*p* < 0.01, and ^*###*^*p* < 0.001 versus the CIA + PBS group

To evaluate the preventive or therapeutic effect of HAR on CIA, RA model was constructed by immunizing DBA/1 mice with bovine CII in CFA and then subjected to CII in IFA on day 21 (Fig. 1A). After the second immunization, the CIA group showed a tendency to lose weight compared to the control group by boosting, but no significant differences were observed between the groups (Fig. 1E). CIA mice exhibited a phenotype of severe swelling, erythema, and joint rigidity of the hind paws, resulting in increased disease incidence (Fig. 1B, C, D). In contrast, in mice administred HAR, the incidence and severity of CIA were significantly reduced before or after the onset of clinical arthritis (Fig. 1B, C, D). Articular destruction and bone damage were confirmed in the hind paws using micro-CT. The destruction in the tarsometatarsal joints, calcaneus, or tarsal bone were reduced in HAR-administered mice compared to PBS-treated mice (Fig. 2A). Examination of microarchitectural characteristics revealed that the total porosity value increased, and the Tb·Pf value decreased along the course of CIA. In micro-CT analysis of the calcaneus, the total porosity value was significantly decreased, and the Tb·Pf value was significantly increased in mice administered HAR before or after the development of clinical arthritis (Fig. 2B). Histological assessment revealed inflammatory cell infiltration, cartilage damage, pannus formation, and bone damage in the ankle joints (Fig. 2C). The ankle joints from the HAR-administered mice showed remarkably decreased inflammation, joint destruction, and bone erosion compared to PBS-treated mice (Fig. 2C). Histological analysis of ankle joints using H&E, safranin O, and toluidine blue staining showed that compared with control, CIA mice induced inflammation, cell infiltration and synovial hyperplasia, which were inhibited by HAR administration (Fig. 2C, D). Next, considering that TRAP is an active marker of osteoclasts, we analyzed TRAP-positive osteoclast in mouse joint sections. As shown Fig. 2C and E, CIA mice had increased TRAP-positive osteoclasts in the bone erosion region compared to control mice, and incontrast, HAR administration significantly dereased them.

### HAR suppresses systemic bone destruction in CIA mice

**Fig. 3:**
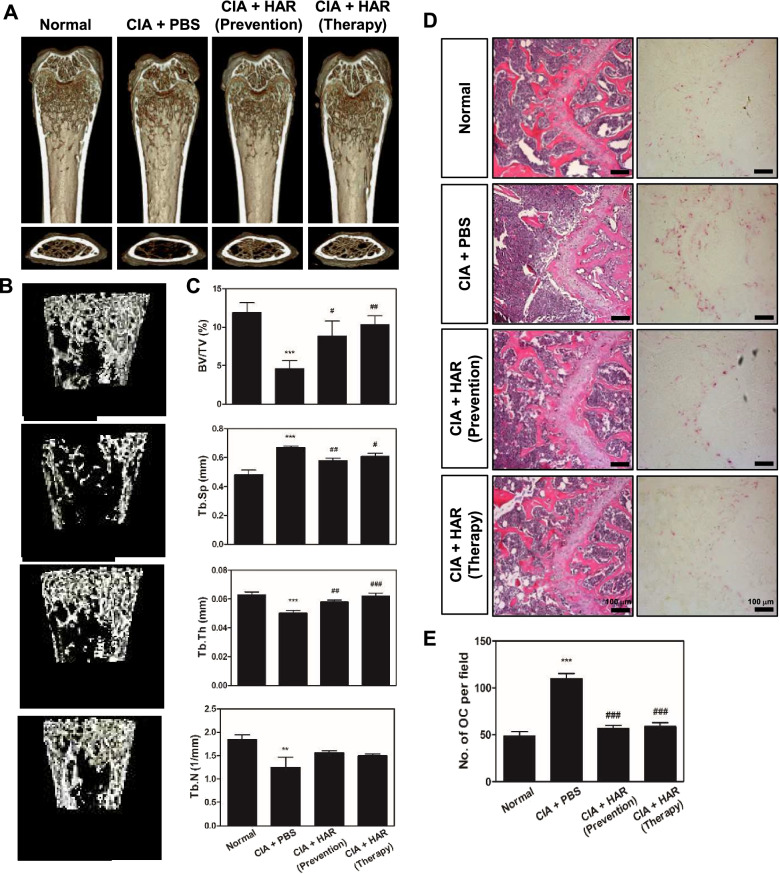
Effect of harpagoside (HAR) on bone erosion, bone destruction, and TRAP formation in collagen-induced arthritis (CIA) mice. **A** Mice were sacrificed on day 42 after the first immunization, and radiographs of the confocal and transverse planes of proximal femur were obtained from micro-CT apparatus. **B** Micro-CT images of specific regions of the trabecular bone for the assessment of various bone parameters. **C** The bone volume per tissue volume (BV/TV), trabecular separation (Tb.Sp), trabecular thickness (Tb,Th), and trabecular number (Tb.N) of femur were determined using the micro-CT data and analyzed by INFINITT-Xelis Software. After micro-CT scans, dissected femurs were fixed, decalcified, embedded, and sectioned. Sections were stained with (**D**) hematoxylin and eosin (H&E) (left) and TRAP (right). Scale bar, 100 μm. **E** The number of osteoclasts per field was counted using the histomorphometric methods. ^**^p < 0.01 and ^***^p < 0.001 versus the normal group; ^*#*^*p* < 0.05, ^*##*^*p* < 0.01, and ^*###*^*p* < 0.001 versus the CIA + PBS group

The inflammatory process in RA adversely affects bone remodeling and shifts the balance towards resorption. To address the effect of HAR on systemic bone loss, we evaluated the proximal femur using micro-CT. A 3-dimensional analysis revealed a loss of trabecular bone following CIA treatment, which was abrogated in mice administered with HAR before or after the development of clinical arthritis (Fig. 3A, B). In addition, histomorphometric measurements of the femur showed protection of the trabecular structure in mice administered with HAR before or after the development of clinical arthritis compared with PBS-treated arthritic control. A significant increase in trabecular bone indices, including BV/TV and Tb. Th, was observed in mice administered with HAR before or after the development of clinical arthritis compared to PBS-treated CIA mice (Fig. 3C). HAR treatment significantly increased Tb. Sp in the CIA mice (Fig. 3C). Furthermore, histological analyses confirmed that treatment with HAR in prophylactic or therapeutic conditions reversed the CIA-mediated loss of the trabecular bone matrix within growth plates and suppressed TRAP-positive osteoclast formation in vivo (Fig. 3D). As anticipated, the number of osteoclasts per visual field was increased in the PBS-treated CIA mice compared to the control, and this increasing trend was significantly blocked by HAR (Fig. 3E).

### HAR inhibits proinflammatory cytokine production and RANKL/OPG ratio

**Fig. 4:**
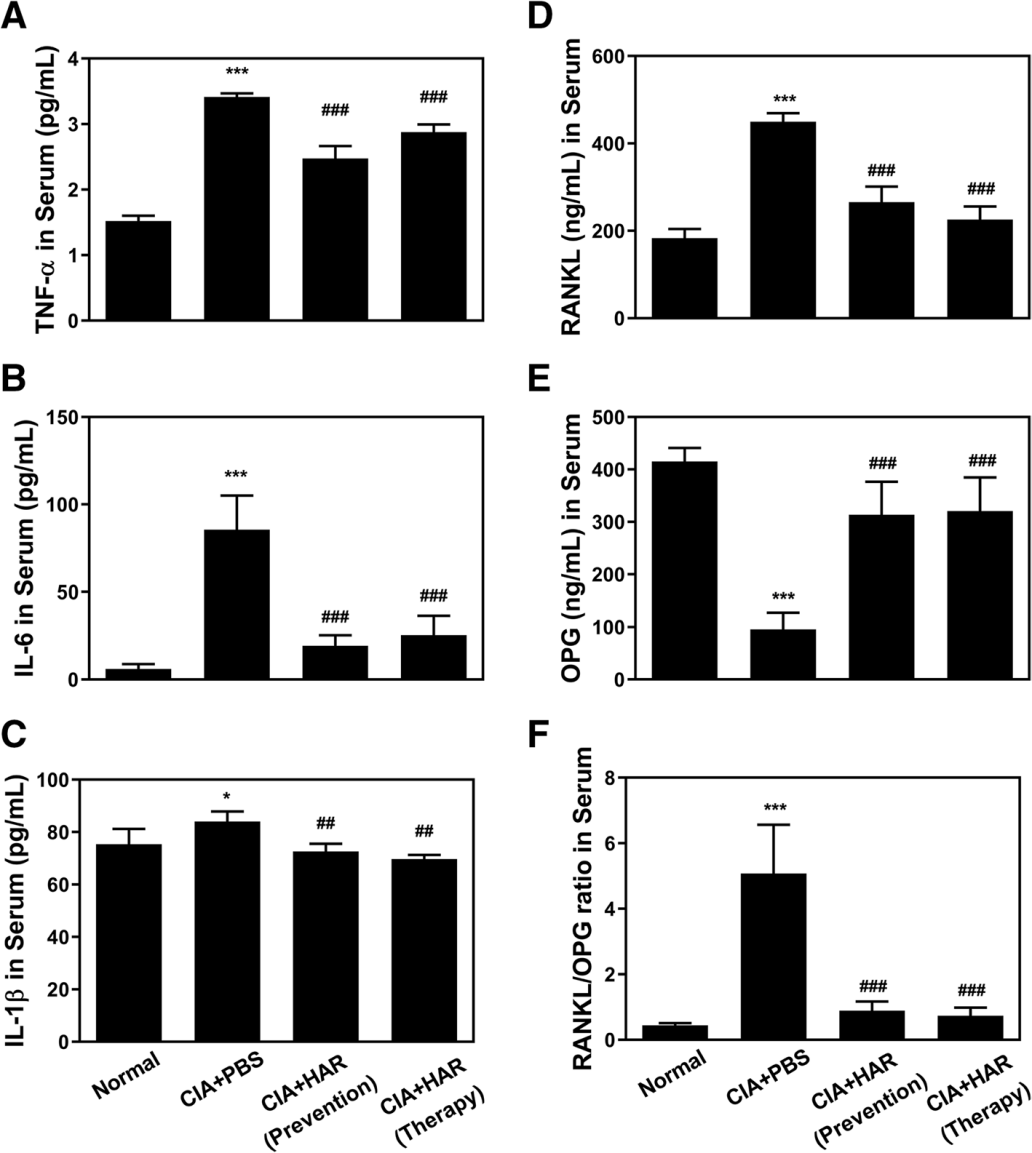
Effect of harpagoside (HAR) on pro-inflammatory mediators and bone markers in serum of collagen-induced arthritis (CIA) mice. Serum was prepared from CIA mice on day 42. The expression levels of (A) TNF-α, (B) IL-6, (C) IL-1β, (D) RANKL, and (E) OPG were determined using murine ELISA assay, and the ratio of (F) RANKL/OPG was calculated. ^*^p < 0.05 and ^***^p < 0.001 versus the normal group; ^*##*^*p* < 0.01 and ^*###*^*p* < 0.001 versus the CIA + PBS group

We next experimented the underlying mechanisms of HAR-induced reduction in the onset and severity of CIA. Since various cytokines are involved in the progression of arthritis, serum cytokine levels obtained on day 42 by ELISA. Increased TNF-α, IL-6, and IL-1β in PBS-treated CIA mice compared to control were observed to be significantly decreased by HAR treatment (Fig. 4A, B, C). In addition, significant increases or decreases in RANKL or OPG expression, respectively, were observed in the serum of PBS-treated CIA mice compared to the control group (Fig. 4D, E). However, HAR-treated mice showed significantly decreased RANKL and increased OPG, thereby decreasing the ratio of RANKL/OPG ratio compared to the CIA group (Fig. 4D, E, F).

### HAR decreases the production of proinflammatory cytokines in TNFα-stimulated arthritic synovial cells

**Fig. 5:**
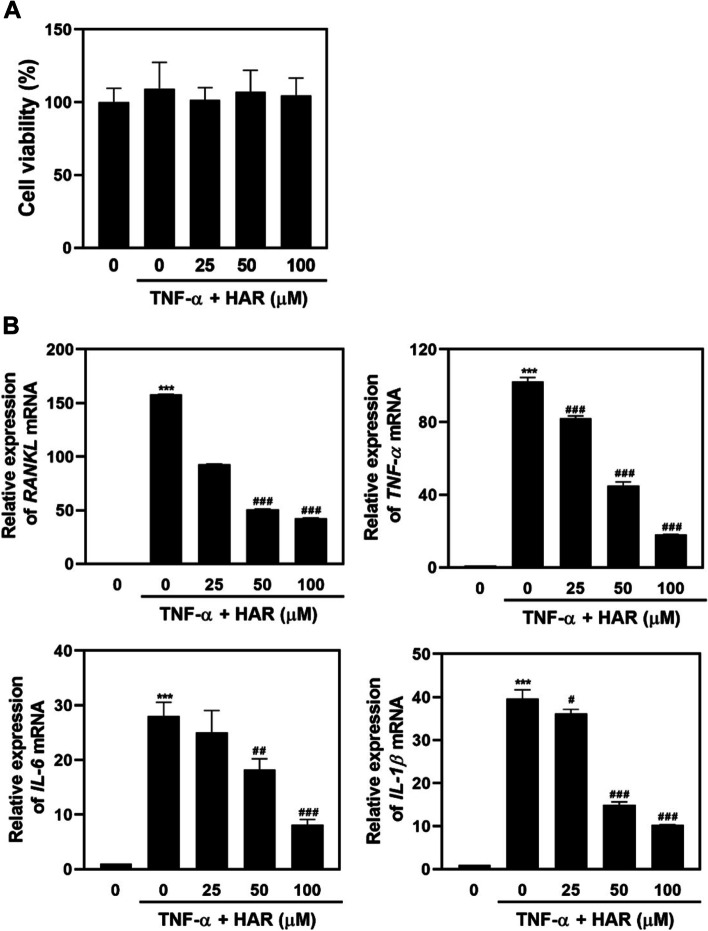
Effect of harpagoside (HAR) on the production of proinflammatory cytokines, RANKL, TNFα, IL-6, and IL-1β in TNFα-stimulated SW982 synovial cells. SW982 synovial cells were treated various doses of HAR (0, 25, 50 and 100 μM) for 1 h and then incubated with TNFα (50 ng/mL) for 24 h. **A** Cell viability was determined using the XTT assay. **B** The total RNA was extracted and real-time RT-PCR was performed to measure the transcripts of *RANKL*, *TNFα*, *IL-6*, and *IL-1β*. The mRNA levels of these genes were normalized to *GAPDH* and represented as fold change over the TNFα-untreated, HAR-untreated cells. ^***^p < 0.001 versus TNFα-untreated, HAR-untreated cells; ^*#*^*p* < 0.05, ^*##*^*p* < 0.01 and ^*###*^*p* < 0.001 versus TNFα-treated, HAR-untreated cells

SW982 synovial cells were treated various doses of HAR (0, 25, 50 and 100 μM) for 1 h and then incubated with TNFα (50 ng/mL) for 24 h, followed by XTT assay for 2 h. HAR at concentrations between 25 and 100 μM showed no evidence of cytotoxicity (Fig. 5A). TNF-α stimulation dramatically upregulateted the mRNA levels of proinflammatory cytokines including *IL-1β*, *IL-6* and *TNF-α*. However, these upregulations were significantly inhibited by HAR treatment (Fig. 5B), which confirmed the anti-inflammatory action of HAR. RANKL has been regarded as the strongest inducer in osteoclast differentiation and activity. In our study, RANKL was significantly suppressed by HAR at the mRNA levels (Fig. 5B), indicating that HAR might play an important protective role in bone destruction.

### HAR inhibits osteoclast formation and bone resorption

**Fig. 6:**
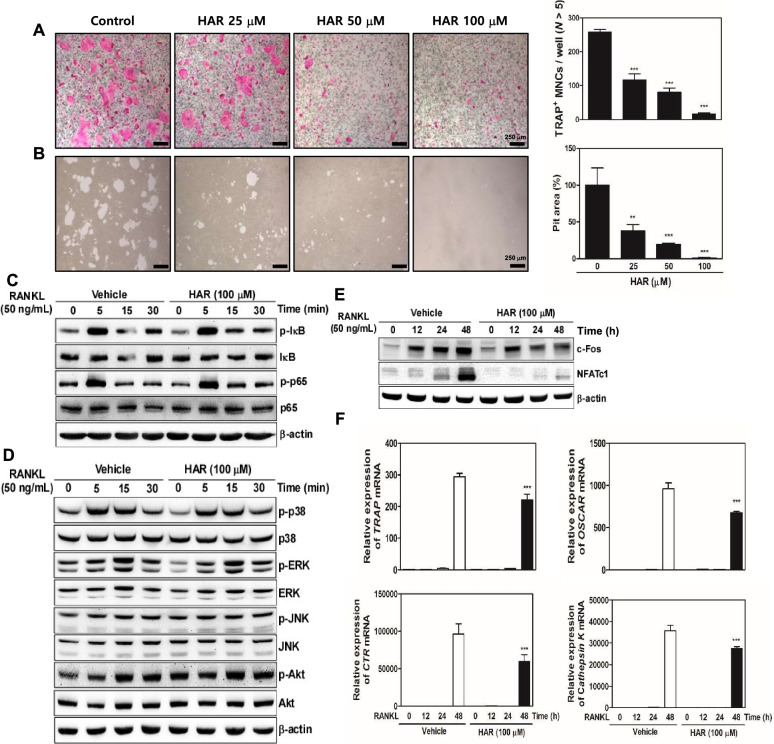
Effect of harpagoside (HAR) on RANKL-induced osteoclast differentiation, bone resorption, and signal pathway in bone marrow macrophages (BMMs). **A** BMMs were cultured for 4 days in the presence of M-CSF (30 ng/mL) and RANKL (50 ng/mL) with HAR (0–100 μM). Then, the cells were fixed with 3.7% formalin, permeabilized with 0.1% Triton X-100, and stained with TRAP. Scale bar, 250 μm. TRAP-positive multi-nucleated osteoclasts (TRAP^+^ MNCs) with more than five nuclei were counted. **B** Mature osteoclasts were seeded on hydroxyapatite-coated plates and treated with the indicated concentrations of HAR for 24 h. The attached cells on the plates were removed and photographed under a light microscope. Pit areas were quantified using Image J. **C**-**E** BMMs were pretreated with HAR (100 μM) or vehicle (DMSO) for 1 h before RANKL (50 ng/mL) stimulation at the indicated times. The cell lysates were analyzed by western blotting with the indicated antibodies. **F** The mRNA expressions of *TRAP*, *OSCAR*, *CTR*, and *Cathepsin K* were analyzed by real-time RT-PCR. ^**^p < 0.01 and ^***^p < 0.001 versus the vehicle

Recent studies have suggested that inflammation-induced systemic bone destruction is a key pathological feature of RA mediated by osteoclasts. Thus, to examine the effect of HAR on RANKL-induced osteoclast differentiation, BMMs were incubated with various concentrations (0, 25, 50, and 100 μM) of HAR in the presence of M-CSF (30 ng/mL) and RANKL (100 ng/mL). The results showed that HAR reduced the formation of TRAP-positive cells in a dose-dependent manner (Fig. 6A). In addition, as a result of confirming bone resorbing activity by seeding mature osteoclasts in the presence or absence of 100 μM HAR on a hydroxyapatite-coated plate, HAR treatment inhibited bone resorption of mature osteoclasts (Fig. 6B). Next, to elucidate the molecular mechanism underlying the HAR-mediated early stage of osteoclastogenesis, we tested the effect of HAR on the phosphorylation of early cellular transducers, including NF-κB, p38, ERK, JNK, and Akt. As shown in Fig. 6C and D, the phosphorylation of early cellular transducers increased by RANKL was not significantly changed by HAR. However, it was confirmed that HAR significantly reduced the expression of c-Fos and NFATc1 increased by RANKL compared to the control group, and subsequently inhibited the expression of osteoclast-specific marker genes, such as *TRAP*, *OSCAR*, *CTR*, and *Cathepsin K* (Fig. 6E, F).

## Discussion

Considering the progression of RA, which causes severe pain in the joint after onset, early diagnosis and treatment of RA are as important as prevention of the disease. For effective treatment of RA, it is important to suppress synovial inflammation in a timely manner as well as prevent bone erosion in the joint area [25, 26]. Since the pathological and immunological characteristics induced by CIA administration are similar to those observed in human RA, the murine CIA model is the most commonly used animal model for RA studies [23, 27]. Therefore, the present study focused on dividing the preventive and therapeutic effects of HAR on the bone destruction induced by CIA. Micro-CT 3D photography, parameter analysis, and visual characteristics revealed that inflammation-mediated bone loss, RA incidence, clinical scores, and swelling of paws were significantly inhibited in both the prevention and therapy groups compared to the control group. In addition, histological analysis showed that HAR decreased the destruction of trabecular bone and the formation of osteoclasts in both prevention and therapy models.

HAR exerts an anti-inflammatory effect by inhibiting the inflammatory stimuli through the suppression of c-Fos/AP-1 activity in osteoarthritis chondrocytes [28]. Additionally, we have previously reported that HAR could suppress RANKL-induced osteoclastogenesis and prevent inflammation-mediated bone loss [22]. In this study, HAR restored bone density in an LPS- but not in an ovariectomy-induced bone loss in vivo [22]. Therefore, HAR is more effective against inflammation-associated bone loss, such as inflammatory osteoporosis and RA, than against hormonal-associated bone loss, including postmenopausal osteoporosis [22]. We hypothesized that HAR could have an excellent preventive and therapeutic effects on bone destruction in a CIA-induced RA mouse model. We identified the molecular mechanisms underlying the beneficial effects of HAR against local or systemic stimuli. HAR has been validated to inhibit arthritis caused by inflammatory cytokines and local and systemic bone loss caused by osteoclasts during chronic inflammatory arthritis in the knee joint histology. As expected, RANKL-induced osteoclast differentiation in BMMs significantly reduced the number of osteoclasts and bone resorption activity in proportion to the concentration of HAR (Fig. 6A, B).

Osteoclast-mediated local bone erosion and destruction in RA is specialized in cells expressing definitive features. The production of RANKL by cells within the inflamed synovium implies a mechanism for osteoclast differentiation and activation in the region of bone erosion [29]. RANKL is a representative stimulator of osteoclast differentiation and function in vitro and in vivo. RANKL induces the expression of osteoclast-specific marker genes, such as *TRAP*, *OSCAR*, *CTR*, and *Cathepsin K*, which can induce osteoclast formation and bone resorption. HAR reduced the formation of TRAP^+^ MNCs but did not affect the early signaling pathway involved in osteoclast differentiation (Fig. 6C, D). In a previous study, HAR inhibited c-Fos and NFATc1 through Syk-Btk-PLCγ2-Ca^2+^ signaling [19]. The results of previous studies and those of our study showed that HAR significantly inhibited the expression levels of c-Fos and NFATc1 (Fig. 6E), subsequently reducing the mRNA expression of *TRAP*, *OSCAR*, *CTR*, and *Cathepsin K* (Fig. 6F).

RA is mediated with an increased production of various of cytokines including TNF-α, IL-1β, and IL-6, leading to joint inflammation and bone erosion that contribute to the pathogenesis of RA. TNF-α, expressed mainly by macrophage and synovial lining cells, and active T cells in RA-inflamed joint, is the predominant pro-inflammatory cytokine and also induces the production of other pro-inflammatory cytokines [30, 31]. Also, activated macrophages and synovial fibroblasts in RA joints are sources of IL-1 production [32]. Blocking the induction of TNF-α and IL-1 in the hTNF.tg mouse model resulted in disease mitigation through inhibition of osteoclast differentiation, synovial inflammation, erosion of bone and cartilage [33]. IL-6 is induced in various cell types including macrophages, fibroblast-like synoviocytes, and chondrocytes in the inflamed RA bone microenvironment [34]. Mice overexpressing IL-6 develop osteopenia as well as severe alterations in trabecular bone microstructure with decreased osteoblasts and increased osteoclast number and activity [35], whereas IL-6 deficient mice suppress inflammation and bone erosion in an antigen-induced arthritis model [36]. Efforts should be made to inhibit the expressions of pro-inflammatory cytokines as well as genes involved cartilage and bone destruction in RA treatment. Our studies revealed that TNF-α stimulation could dramatically upregulate the mRNA levels of pro-inflammatory cytokines including *IL-1β*, *IL-6* and *TNF-α*. However, these upregulations were significantly inhibited by HAR treatment, which further confirmed the anti-inflammatory action of HAR (Fig. 5B). RANKL was significantly suppressed by HAR in TNF*α*-stimulated SW982 synovial cells (Fig. 5B) and bone resorption was inhibited through reduced expression of c-Fos/NFATc1 and marker genes related to osteoclastogenesis (Fig. 6F), indicating that HAR might play an important protective role in bone destruction. Furthermore, in CIA model, the relative expression ratios of RANKL/OPG and the secretion levels of TNF-α, IL-1β, and IL-6 were diminished because HAR inhibited osteoclast differentiation and formation (Fig. 4). Based on these results, it was confirmed that HAR controlled the inflammatory activated cells leading to RA by exerting an inhibitory effect on the underlying mechanisms of osteoclast formation and inhibiting the production of pro-inflammatory cytokines, which are crucial factors in bone destruction. Taken together, these data indicated that HAR might play an inhibitory action on RA via multiple targets, and further studies are needed to clarify its direct mechanism and binding sites, by the action of c-Fos and NFATc1 inhibitors and comparison of the effect of HAR and positive control such as denosumab.

## Conclusion

Rheumatoid arthritis is accompanied by pathological conditions that promote bone absorption and reduce bone regeneration. It would be effective to use drugs that can prevent and treat local immunity and systemic bone loss to reduce the risk factors of fractures during illness. Taken together, HAR effectively inhibited the incidence and symptoms in CIA-induced RA mice and effectively suppressed bone loss caused by chronic autoimmune diseases, thereby effectively defending against inflammatory arthritis and bone diseases such as osteopenia and osteoporosis. HAR might be used in the prevention and therapy of RA based on its amelioration of synovitis and its inhibitory and preventive effects on joint bone destruction.

## Supplementary Information

**Additional file 1.**

## Data Availability

All the data generated and analyzed in this study are mentioned in this manuscript.

## Abbreviations

RA: Rheumatoid arthritis
HAR: Harpagoside
CIA: Type 2 collagen-induced arthritis
IL: Interleukin
RANKL: Receptor activator of NF-κB ligand
TNF: Tumor necrosis factor
NSAIDs: Nonsteroidal anti-inflammatory drugs
CII: Bovine type II collagen
CFA: Complete Freund’s adjuvant
IFA: Freund’s incomplete adjuvant
LPS: Lipopolysaccharide
micro-CT: Micro-computed tomography
Tb·Pf: Trabecular pattern factor
BV/TV: Trabecular bone volume/total volume
Tb·Th: Trabecular thickness
Tb·Sp: Trabecular separation
Tb·N: Trabecular number
H&E: Hematoxylin and eosin
TRAP: Tartrate-resistant acid phosphatase
BMMs: Bone marrow macrophages
BMCs: Bone marrow cells
α-MEM: α-minimum essential medium
M-CSF: Macrophage colony-stimulating factor
Real-Time RT-PCR: Quantitative real-time reverse transcription polymerase chain reaction
OSCAR: Osteoclast-associated receptor
CTR: Calcitonin receptor
GAPDH: Glyceraldehyde-3-phosphate dehydrogenase
Ct: Cycle threshold
OPG: Osteoprotegerin
ELISA: Enzyme-linked immunosorbent assay
SD: Standard deviation
SE: Standard error
