# Supplementary material for: Harpagoside attenuates local bone Erosion and systemic osteoporosis in collagen-induced arthritis in mice
Source: BMC Complement Med Ther. 2022 Aug 10;22:214. doi: 10.1186/s12906-022-03694-y (PMC9364518; doi:10.1186/s12906-022-03694-y)
Supplement: Supplementary file 1 — Additional file 1. [file 12906_2022_3694_MOESM1_ESM.zip › 1-Supplementary Figures-Western blot-raw data.pdf]

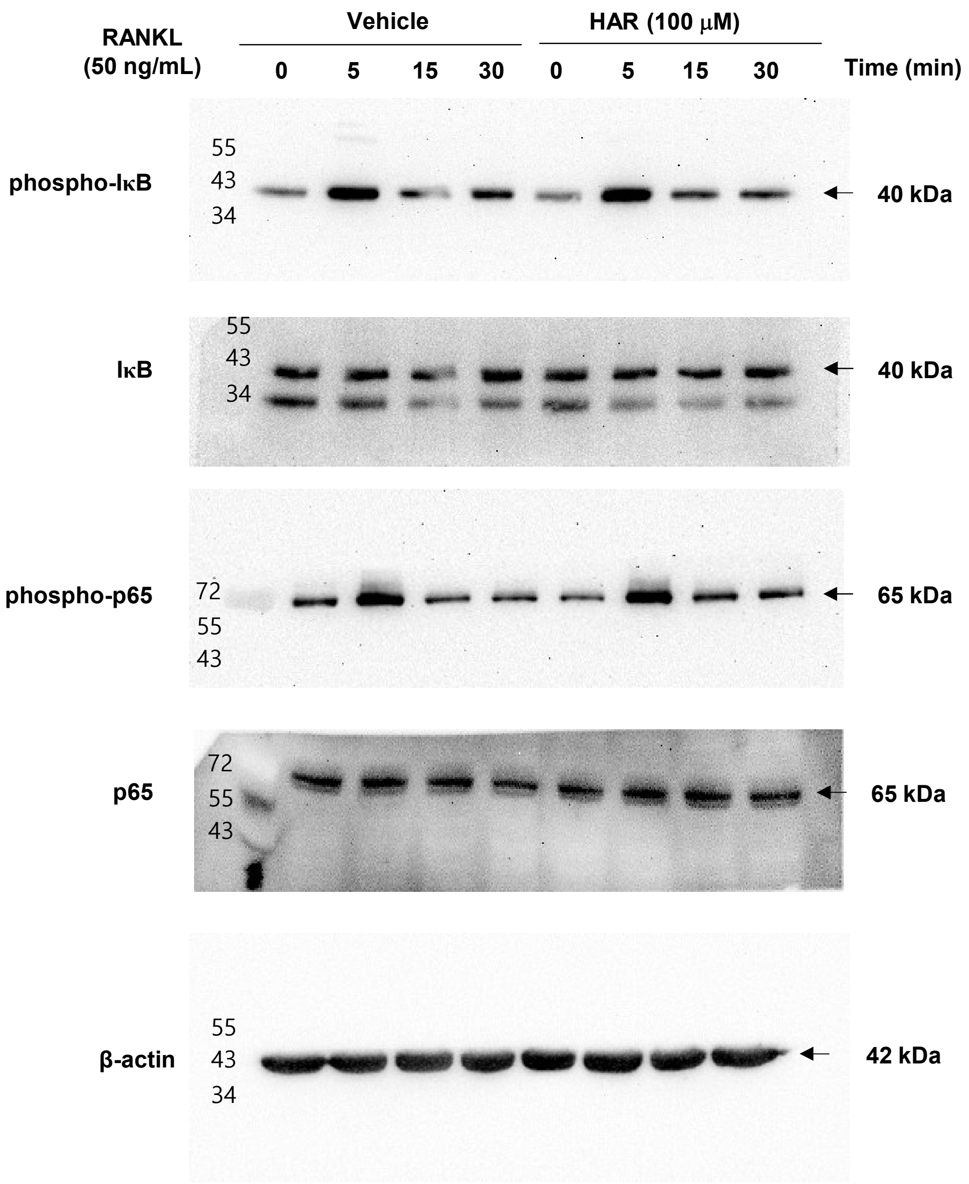

Supplementary Fig. 1. Raw data of western blot results in Fig. 4C. BMMs were pretreated with HAR (100 μM) or vehicle (DMSO) for 1 h before RANKL (50 ng/mL) stimulation at the indicated times. The cell lysates were analyzed by western blotting with the phospho-IκB, IκB, phospho-p65, p65, and β-actin antibodies.

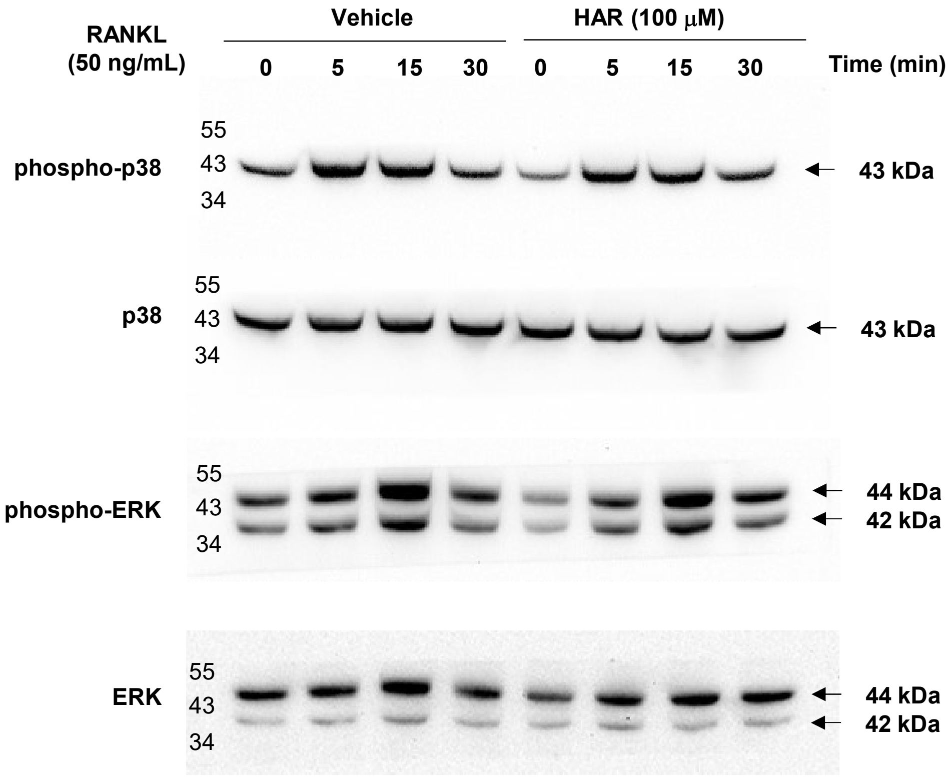

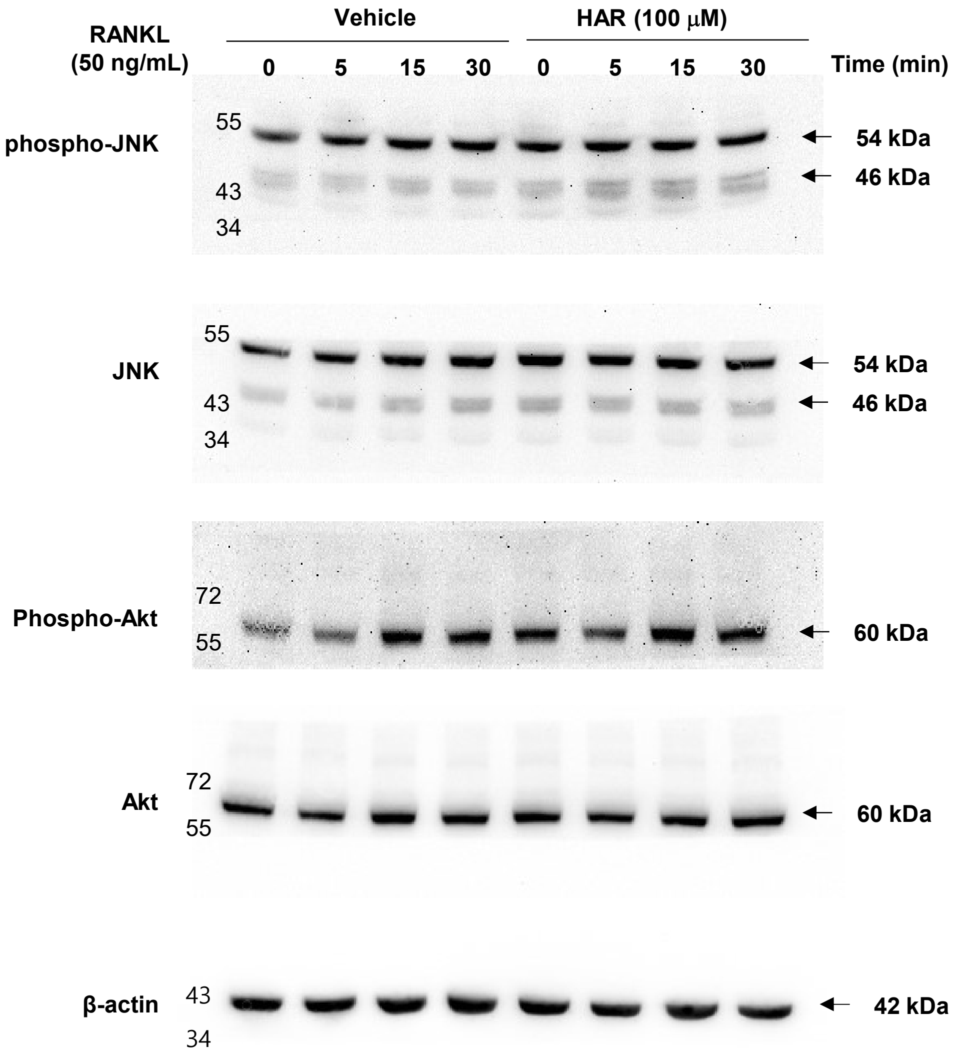

Supplementary Fig. 2. Raw data of western blot results in Fig. 4D. BMMs were pretreated with HAR (100 μM) or vehicle (DMSO) for 1 h before RANKL (50 ng/mL) stimulation at the indicated times. The cell lysates were analyzed by western blotting with the phospho-p38, p38, phospho-ERK, ERK, phospho-JNK, JNK, phospho-Akt, Akt, and β-actin antibodies.

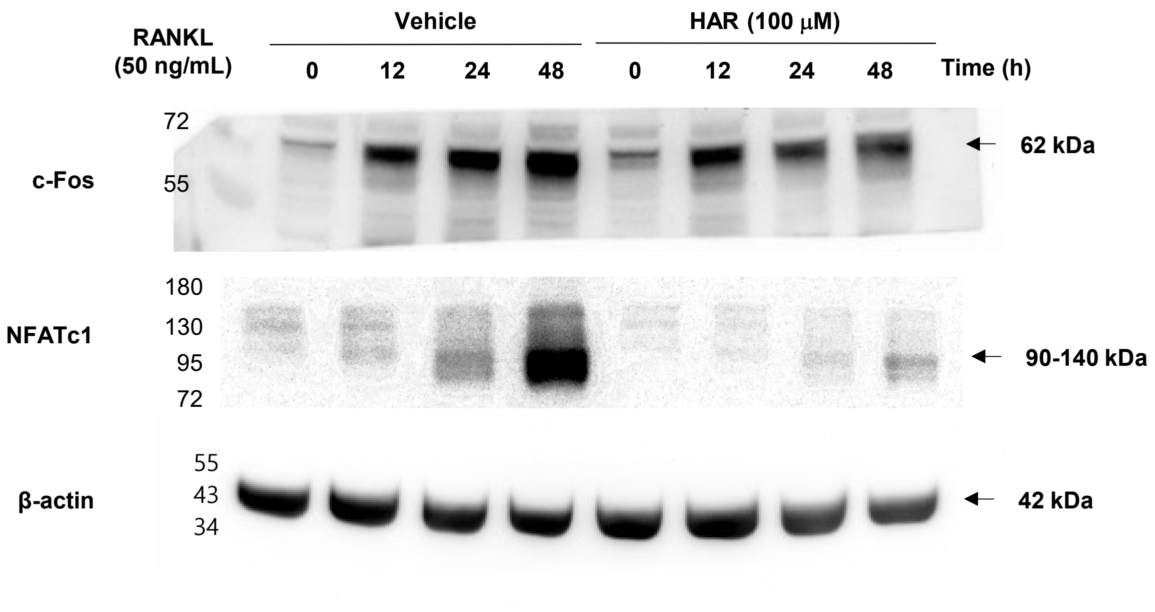

Supplementary Fig. 3. Raw data of western blot results in Fig. 4E.
BMMs were pretreated with HAR (100 μM) or vehicle (DMSO) for 1 h before RANKL (50 ng/mL) stimulation at the indicated times. The cell lysates were analyzed by western blotting with the c-Fos, NFATc1, and β-actin antibodies.
