# Supplementary figures and images for: Harpagoside attenuates local bone Erosion and systemic osteoporosis in collagen-induced arthritis in mice
Source: BMC Complement Med Ther. 2022 Aug 10;22:214. doi: 10.1186/s12906-022-03694-y (PMC9364518; doi:10.1186/s12906-022-03694-y)

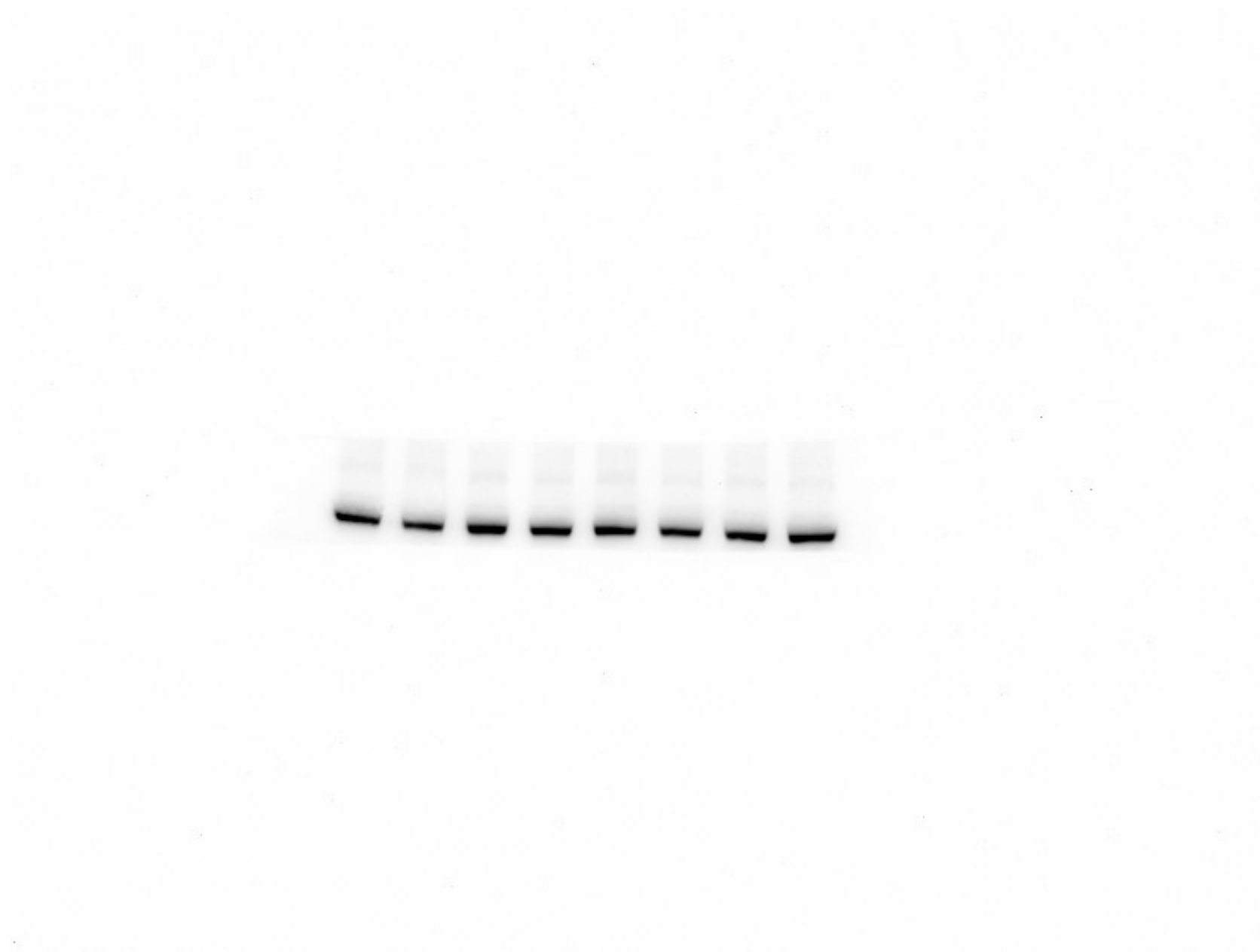

Supplement: Supplementary file 1 — Additional file 1. [file 12906_2022_3694_MOESM1_ESM.zip › 1-Supplementary Fig.2-Akt.pdf]

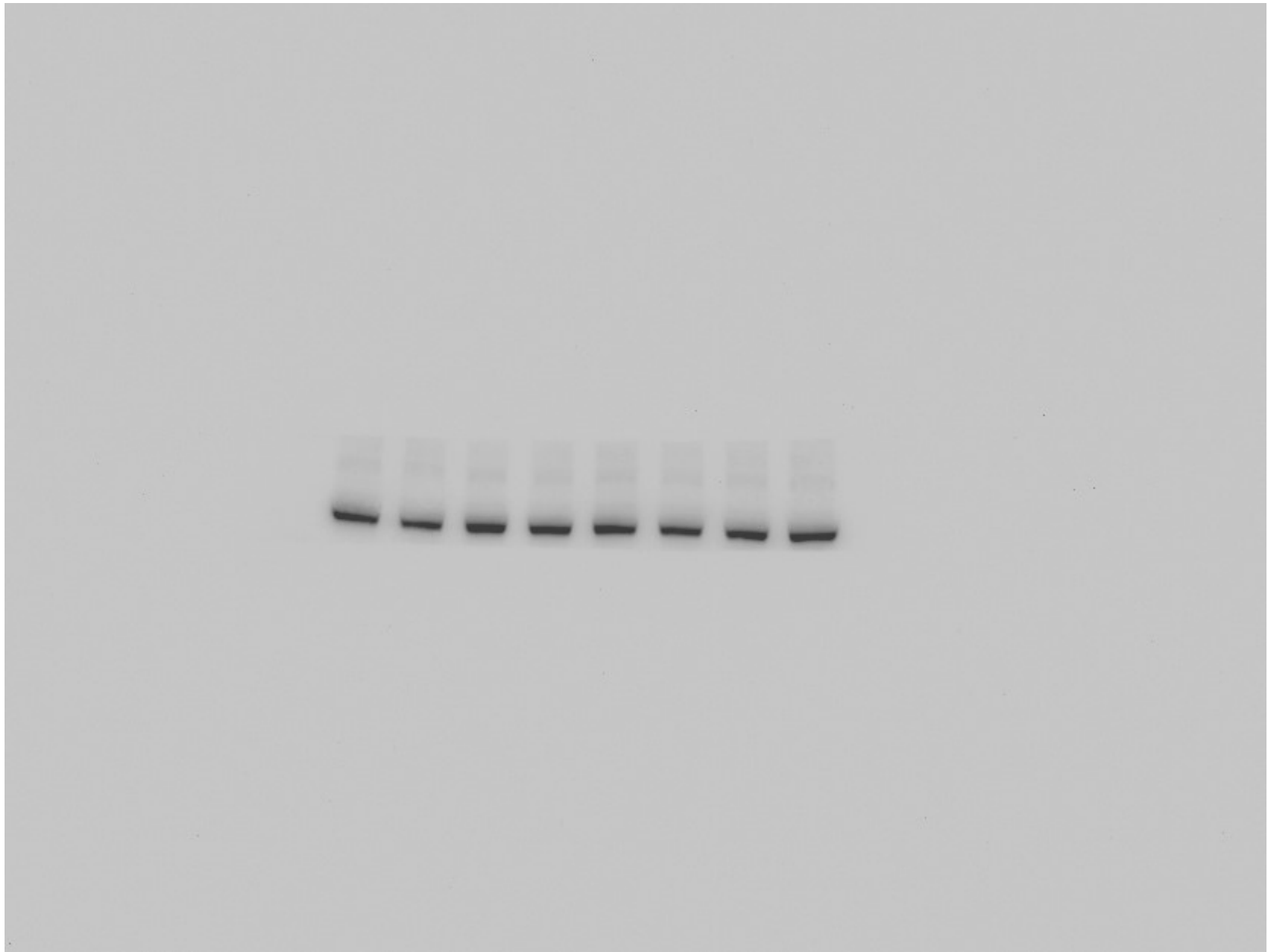

Supplement: Supplementary file 1 — Additional file 1. [file 12906_2022_3694_MOESM1_ESM.zip › Supplementary Fig. 2-Akt-Edited image.pdf]

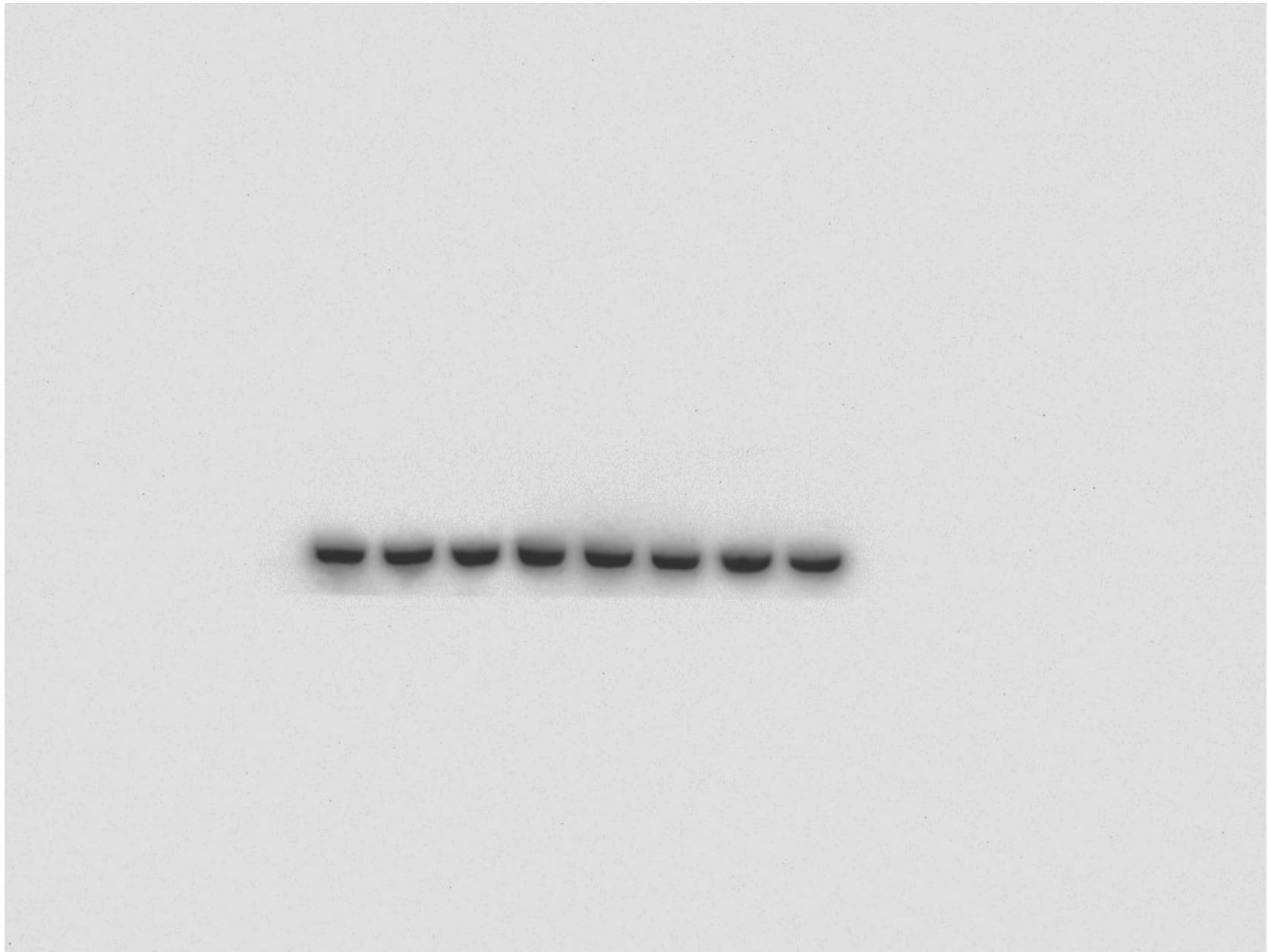

Supplement: Supplementary file 1 — Additional file 1. [file 12906_2022_3694_MOESM1_ESM.zip › Supplementary Fig. 2-beta-actin-Edited image.pdf]

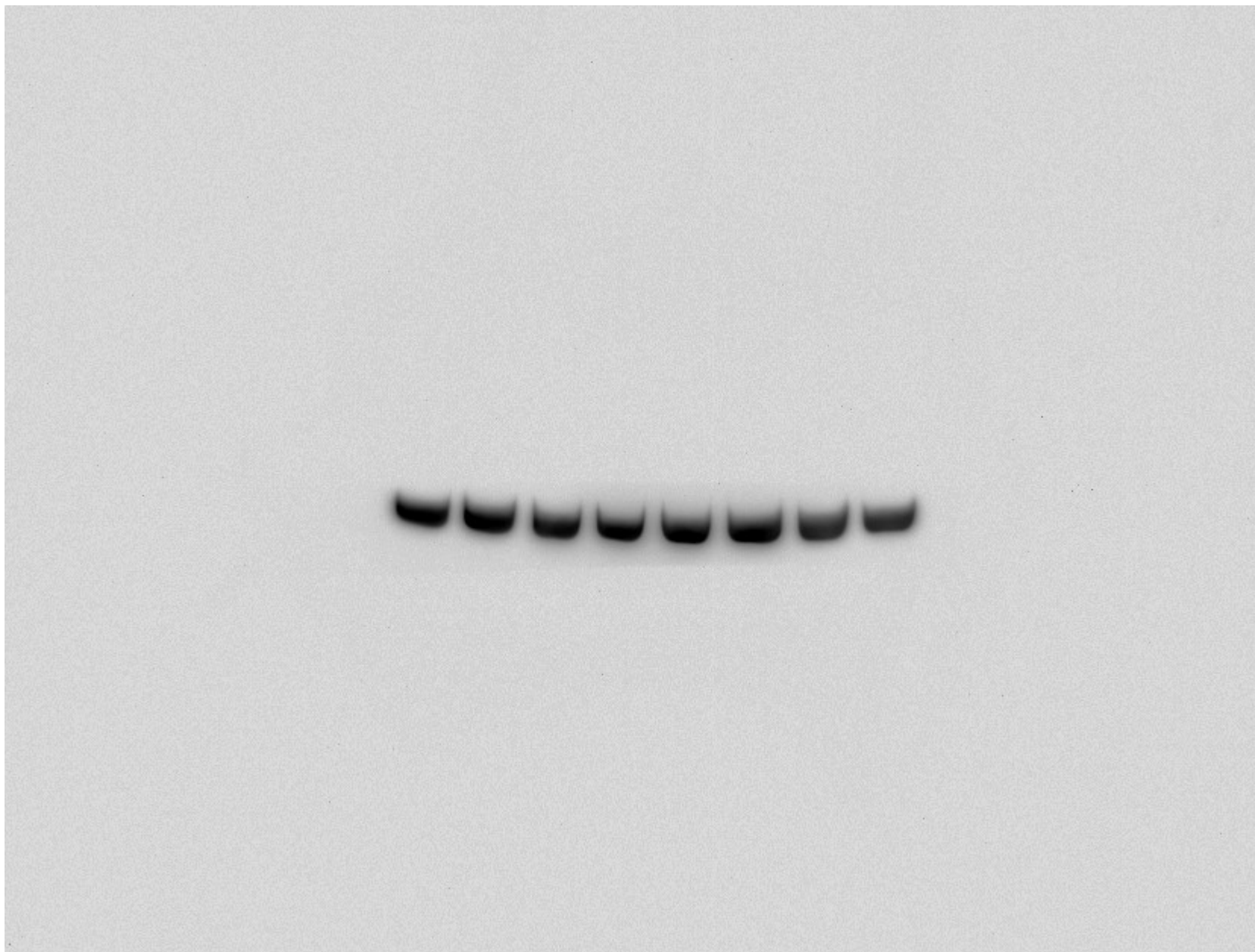

Supplement: Supplementary file 1 — Additional file 1. [file 12906_2022_3694_MOESM1_ESM.zip › Supplementary Fig. 3-beta-actin-Edited image.pdf]

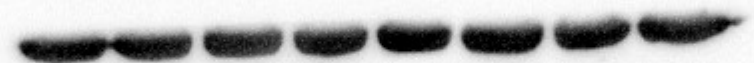

Supplement: Supplementary file 1 — Additional file 1. [file 12906_2022_3694_MOESM1_ESM.zip › Supplementary Fig.1-beta-actin.pdf]

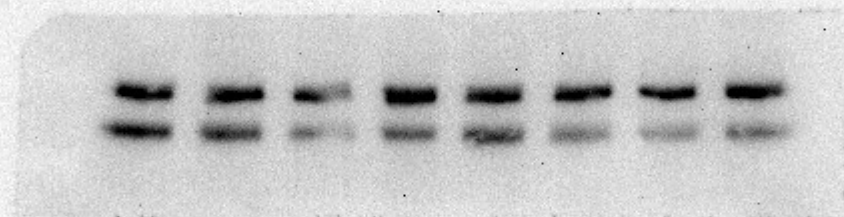

Supplement: Supplementary file 1 — Additional file 1. [file 12906_2022_3694_MOESM1_ESM.zip › Supplementary Fig.1-IkB.pdf]

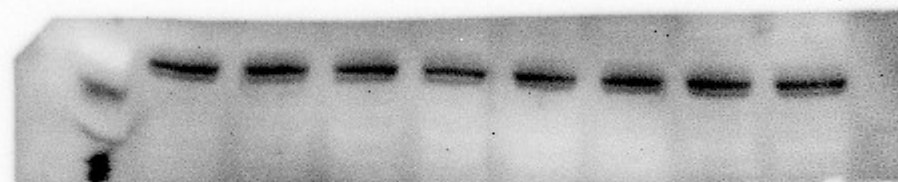

Supplement: Supplementary file 1 — Additional file 1. [file 12906_2022_3694_MOESM1_ESM.zip › Supplementary Fig.1-p65.pdf]

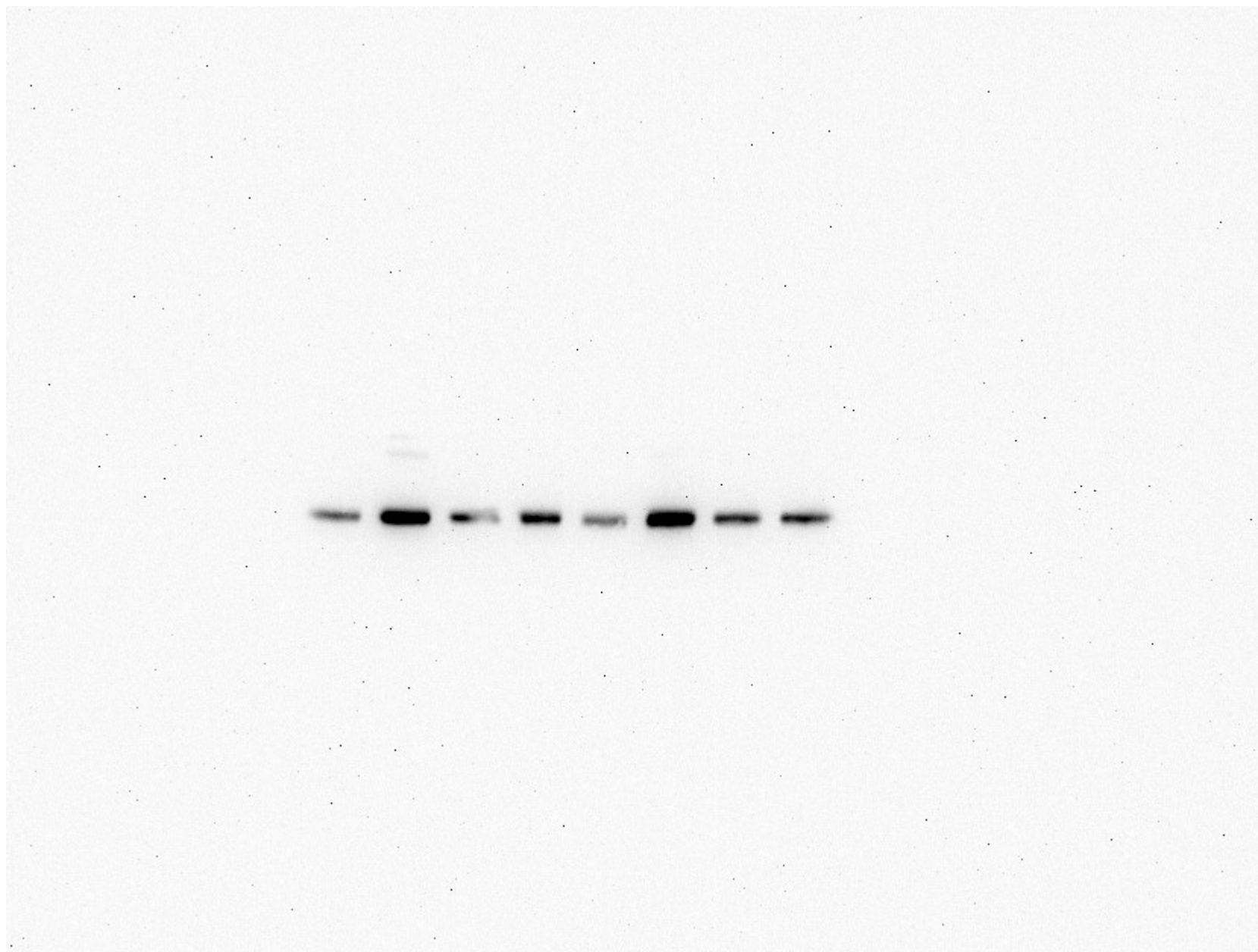

Supplement: Supplementary file 1 — Additional file 1. [file 12906_2022_3694_MOESM1_ESM.zip › Supplementary Fig.1-phospho-IkB.pdf]

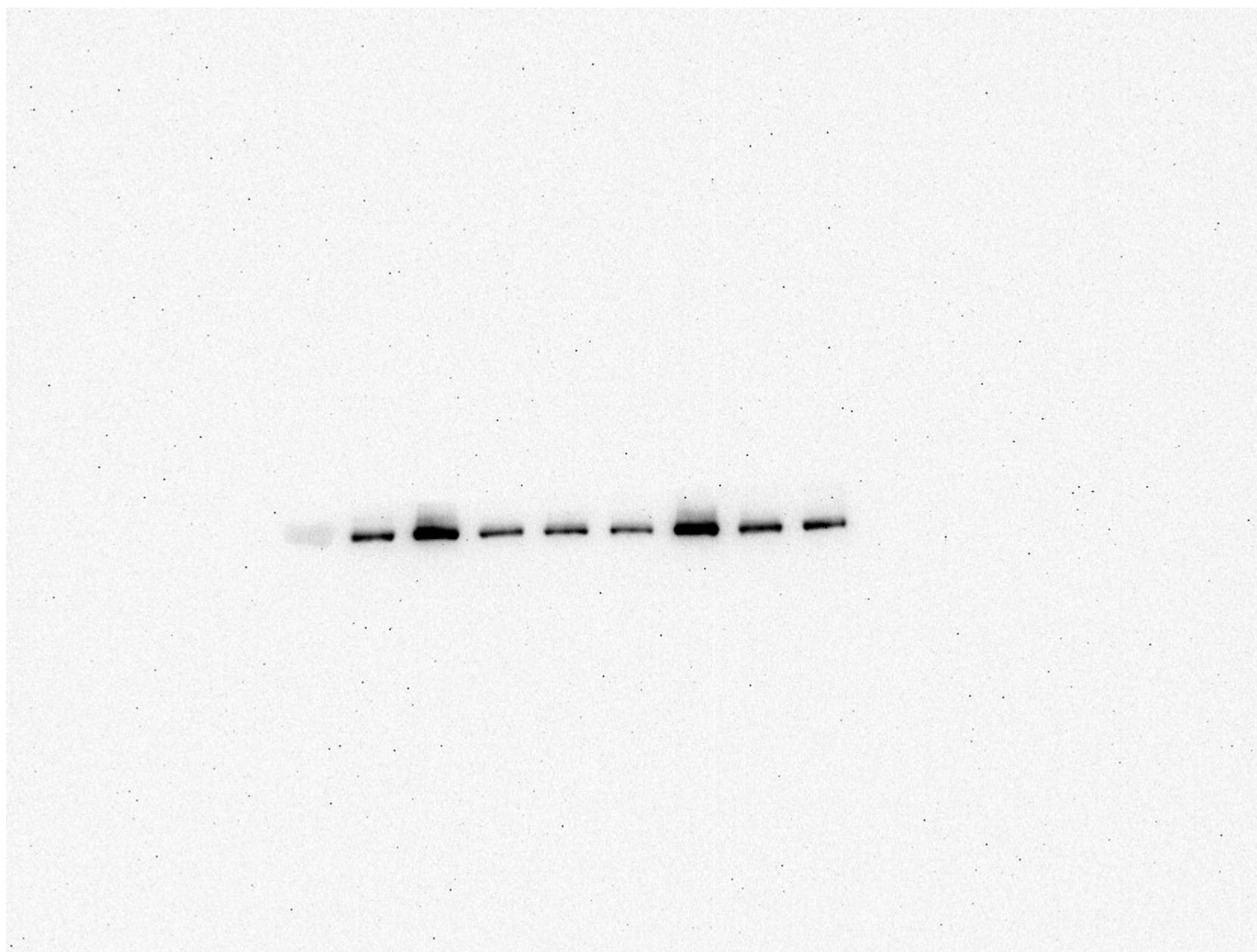

Supplement: Supplementary file 1 — Additional file 1. [file 12906_2022_3694_MOESM1_ESM.zip › Supplementary Fig.1-phospho-p65.pdf]

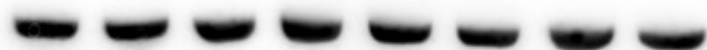

Supplement: Supplementary file 1 — Additional file 1. [file 12906_2022_3694_MOESM1_ESM.zip › Supplementary Fig.2-beta-actin.pdf]

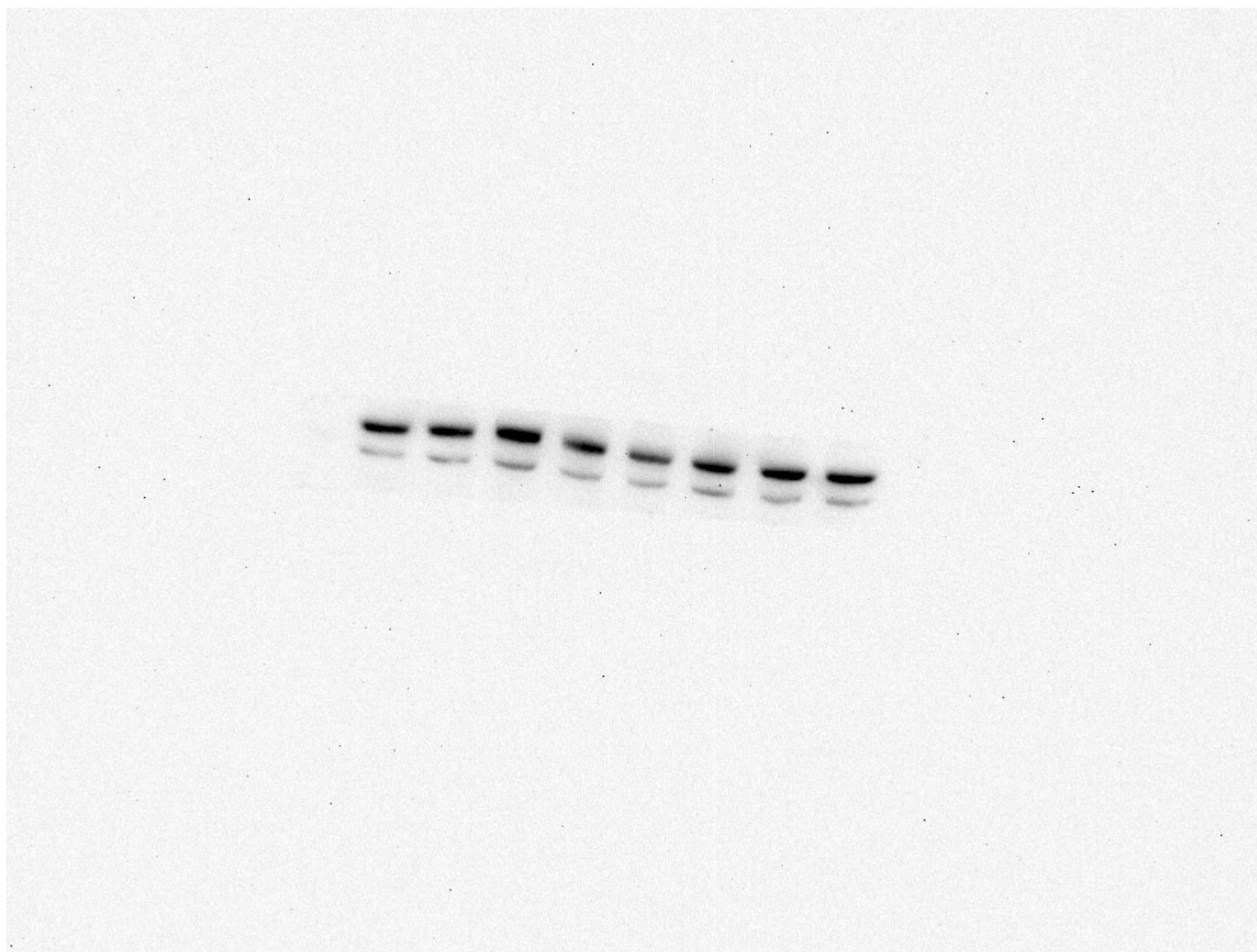

Supplement: Supplementary file 1 — Additional file 1. [file 12906_2022_3694_MOESM1_ESM.zip › Supplementary Fig.2-ERK.pdf]

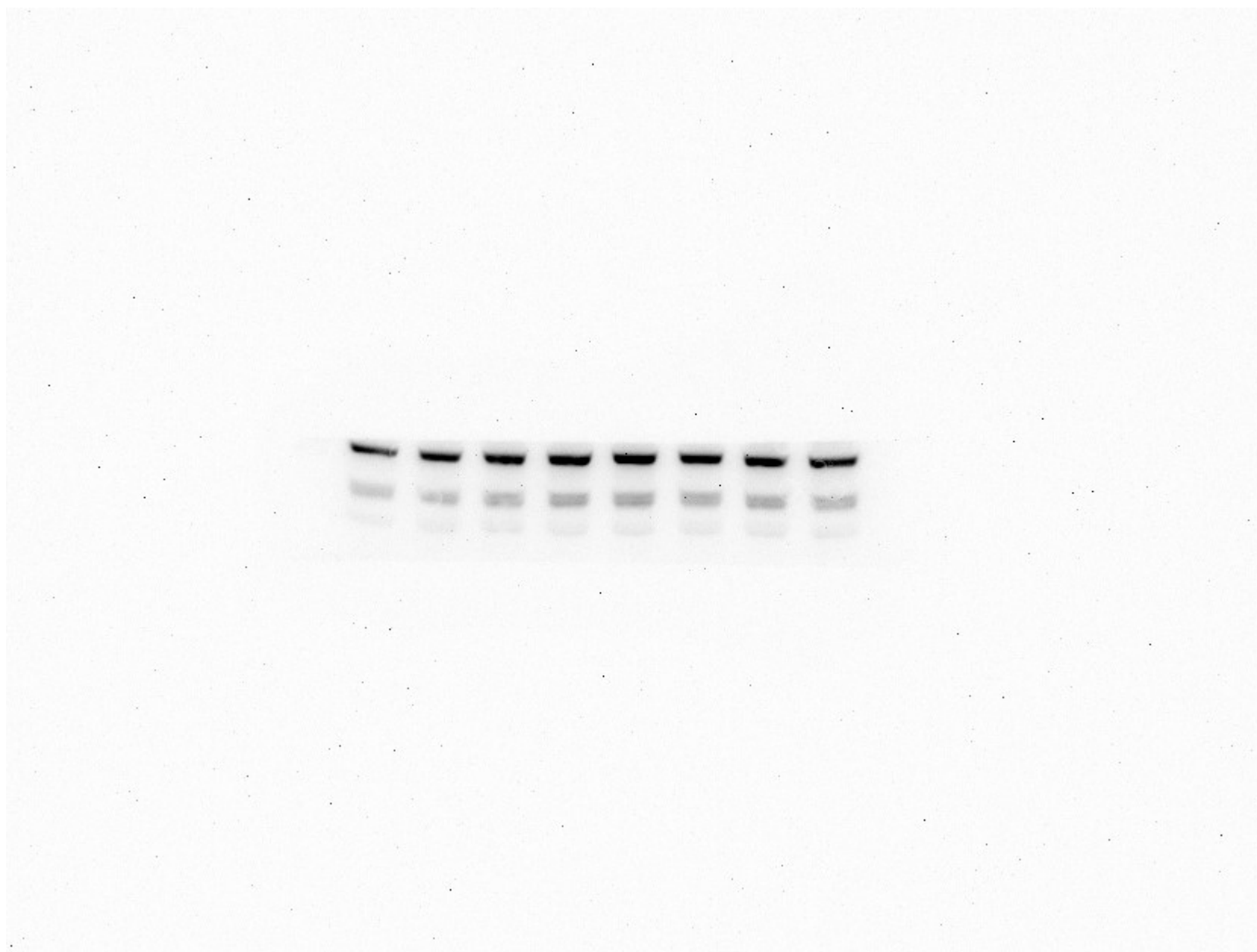

Supplement: Supplementary file 1 — Additional file 1. [file 12906_2022_3694_MOESM1_ESM.zip › Supplementary Fig.2-JNK.pdf]

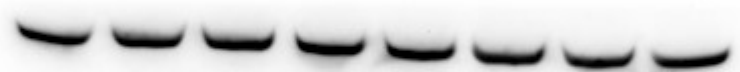

Supplement: Supplementary file 1 — Additional file 1. [file 12906_2022_3694_MOESM1_ESM.zip › Supplementary Fig.2-p38.pdf]

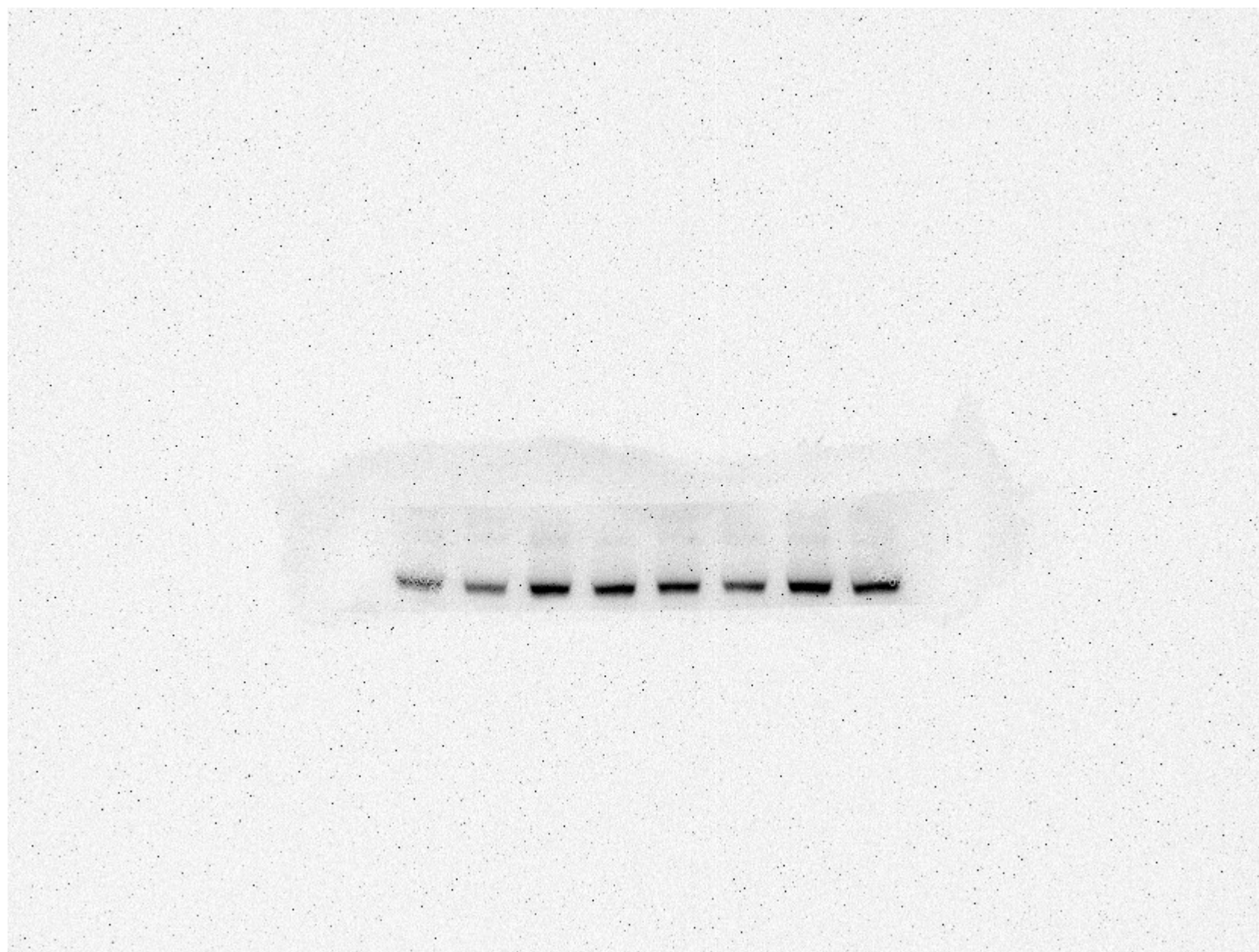

Supplement: Supplementary file 1 — Additional file 1. [file 12906_2022_3694_MOESM1_ESM.zip › Supplementary Fig.2-phospho-Akt.pdf]

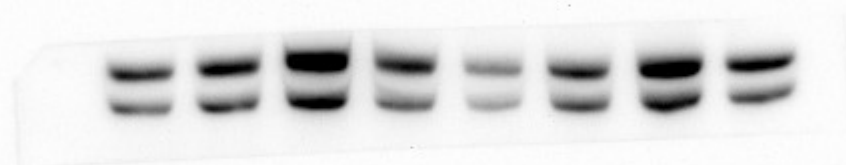

Supplement: Supplementary file 1 — Additional file 1. [file 12906_2022_3694_MOESM1_ESM.zip › Supplementary Fig.2-phospho-ERK.pdf]

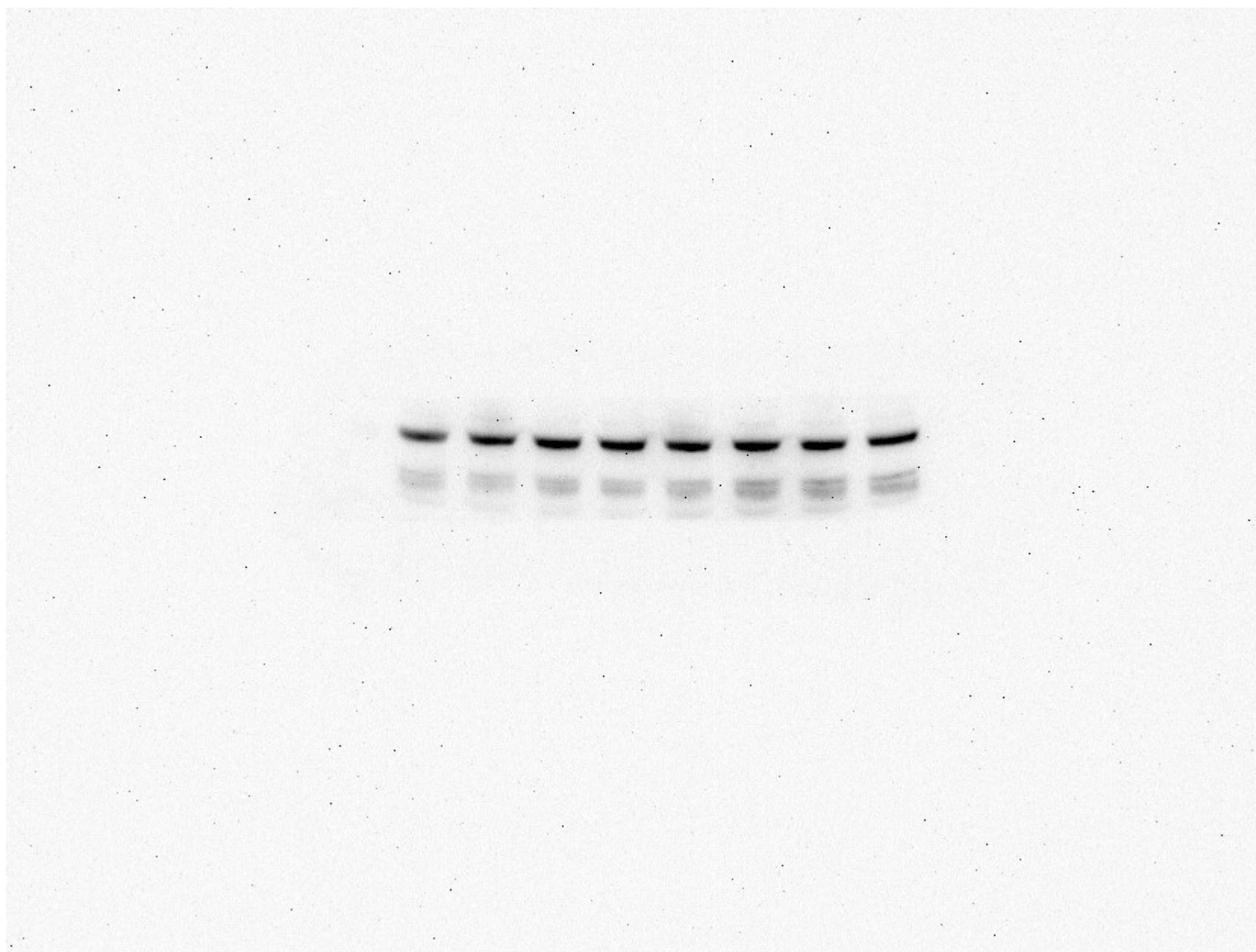

Supplement: Supplementary file 1 — Additional file 1. [file 12906_2022_3694_MOESM1_ESM.zip › Supplementary Fig.2-phospho-JNK.pdf]

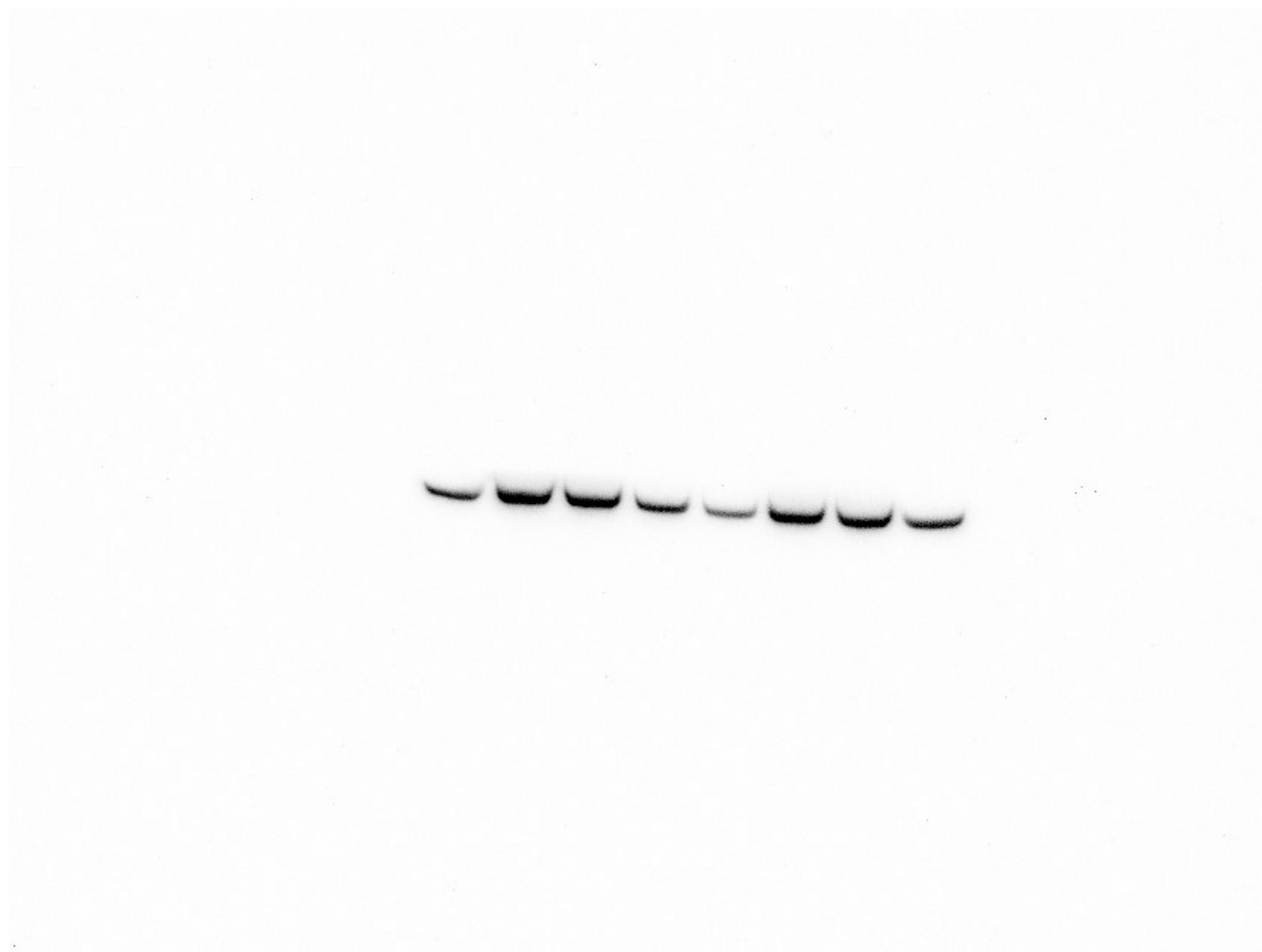

Supplement: Supplementary file 1 — Additional file 1. [file 12906_2022_3694_MOESM1_ESM.zip › Supplementary Fig.2-phospho-p38.pdf]

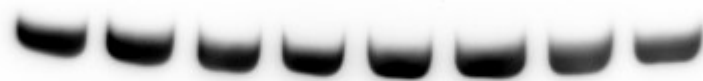

Supplement: Supplementary file 1 — Additional file 1. [file 12906_2022_3694_MOESM1_ESM.zip › Supplementary Fig.3-beta-actin.pdf]

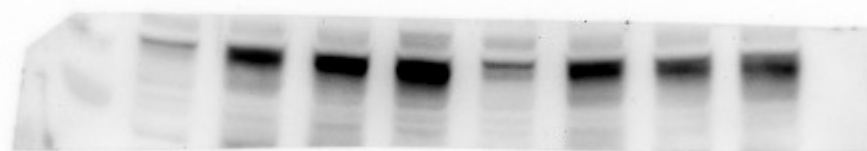

Supplement: Supplementary file 1 — Additional file 1. [file 12906_2022_3694_MOESM1_ESM.zip › Supplementary Fig.3-c-Fos.pdf]

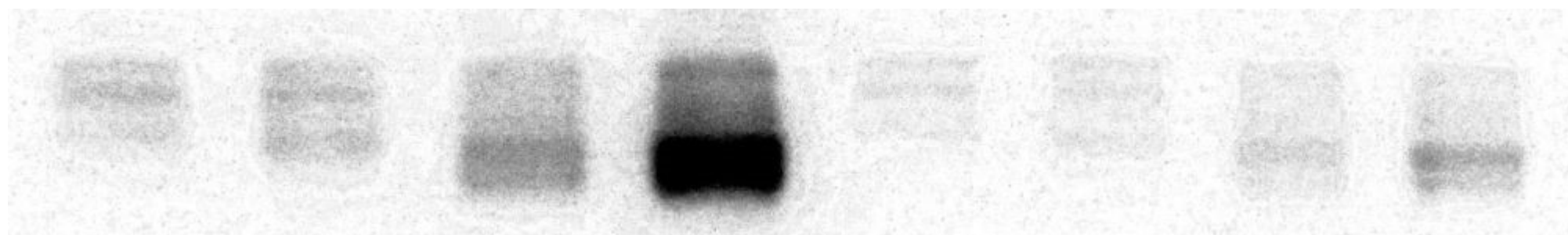

Supplement: Supplementary file 1 — Additional file 1. [file 12906_2022_3694_MOESM1_ESM.zip › Supplementary Fig.3-NFATc1.pdf]
